# AIM2 in tregs restrains anti-PD-1 efficacy by reinforcing immune suppression in MC38 colorectal cancer

**DOI:** 10.1016/j.isci.2026.116082

**Published:** 2026-09-24

**Authors:** Nuo Wang, Hongwei Jiang, Zhang Fang, Jiajin Ma, Hui Ma, Bin Xu, Lujun Chen, Shaoxian Wu, Xiao Zheng

**Affiliations:** 1 Department of Tumor Biological Treatment, The Third Affiliated Hospital of Soochow University, Changzhou, Jiangsu 213003, China; 2 Jiangsu Engineering Research Center for Tumor Immunotherapy, Changzhou, Jiangsu 213003, China; 3 Institute for Cell Therapy of Soochow University, Changzhou, Jiangsu 213003, China; 4 Jiangsu Yize Biological Technology Co., LTD, Changzhou Science and Education Town, Changzhou, Jiangsu 213000, China

**Keywords:** AIM2, regulatory T cells, immune checkpoint inhibitor, tumor microenvironment

## Abstract

Regulatory T cells (Tregs) maintain immune homeostasis but also constrain antitumor immunity. Here, we show that the DNA sensor AIM2 sustains immunosuppressive programs in Tregs and limits responses to immune checkpoint blockade. Using an MC38 colorectal cancer model, Treg specific deletion of AIM2 enhanced the efficacy of PD1 blockade. This effect was associated with reduced effector Tregs and immunosuppressive myeloid populations, alongside increased cytotoxic activity of CD8 T cells. AIM2 deficient Tregs exhibited diminished suppressive programs and enhanced interferon responsive signatures, accompanied by broad remodeling of tumor immune interactions. These findings identify AIM2 as a key regulator of Treg mediated immune suppression and suggest that targeting AIM2 may improve the efficacy of cancer immunotherapy.

## Highlights

• AIM2 sustains immunosuppressive programs in tumor-infiltrating regulatory T cells
• Treg-specific AIM2 deletion enhances response to PD-1 blockade and CD8^+^ T cell activity
• Loss of AIM2 in Tregs reshapes the tumor microenvironment, including myeloid polarization

## Graphical abstract

**Figure:**
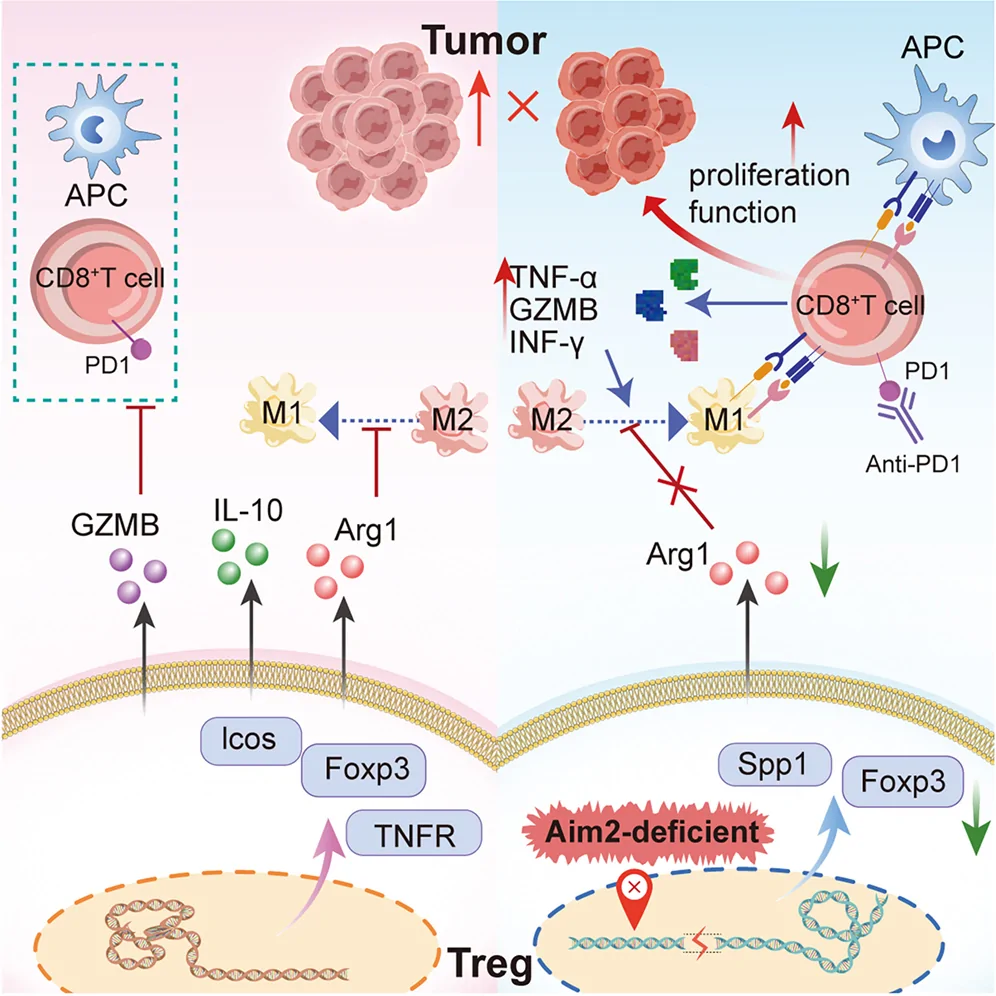

## Teaser

Molecular biology; Immunology; Cell biology; Cancer

## Introduction

Cancer remains a formidable global health challenge, posing a persistent threat to human life and well-being. Although immune checkpoint inhibitors (ICIs) have marked a breakthrough in cancer immunotherapy, their clinical benefits remain limited to a subset of patients, with durable responses still elusive for many. Alarmingly, monotherapy with ICIs has been linked to an elevated risk of early mortality compared to conventional treatment modalities.^1^^,^^2^^,^^3^ In response, a large-scale international effort is underway, encompassing roughly 3,300 clinical trials and involving over half a million participants, all aimed at unlocking the full therapeutic promise of the programmed death-1/programmed death-ligand 1 (PD-1/PD-L1) pathway.^4^ While some trials are expected to yield favorable results for specific patient subgroups, it is increasingly evident that single-agent checkpoint inhibitor is insufficient. A combinatorial approach that integrates additional therapeutic strategies is urgently needed to meaningfully reduce cancer-related morbidity and mortality.

Regulatory T cells (Tregs) are vital for maintaining immune tolerance and preventing autoimmune activation. Within the tumor microenvironment (TME), however, Tregs play a paradoxical role, suppressing antitumor immunity by inhibiting the activation and function of effector T cells.^5^^,^^6^ Their immunoregulatory actions, though physiologically beneficial in preventing immune overactivation, are hijacked by tumors to create an immune-privileged niche that favors tumor progression. The interplay between PD-1/PD-L1 signaling and Treg-mediated suppression is a critical mechanism by which tumor-infiltrating immune cells evade immune surveillance and mount ineffective responses.^7^^,^^8^^,^^9^^,^^10^ High intratumoral Tregs infiltration has been consistently associated with dampened anti-cancer immunity and poor clinical prognosis across multiple malignancies.^11^ Moreover, anti-PD-1 therapy has been shown to enrich CD103^+^ Tregs with enhanced lipid metabolic activity within the TME, where they further constrain the function of CD8^+^ T cells and limit the efficacy of ICIs.^12^ Recent advances have highlighted the therapeutic potential of selectively targeting key molecular pathways within Tregs. Interventions against Treg-intrinsic molecules such as IKAROS,^13^ IFITM,^14^ LTα1β2,^15^ and SOAT2,^16^ as well as bispecific antibodies like NSWh7216 (targeting CD25 and TIGIT),^17^ have demonstrated encouraging preclinical and translational results. These findings collectively underscore the promise of reprogramming Tregs functionality as a strategy to potentiate ICIs therapy and reinvigorate antitumor immunity.

Inflammasomes are pivotal components of the innate immune system, functioning as intracellular sentinels that detect pathogen-associated molecular patterns and initiate inflammatory responses through programmed cell death. Beyond their role in host defense, inflammasomes contribute significantly to tissue homeostasis, inflammatory pathologies, and tumorigenesis.^18^^,^^19^ One such inflammasome sensor, absent in melanoma 2 (AIM2), was originally identified as a tumor suppressor,^20^ but has since been characterized as a cytoplasmic receptor for double-stranded DNA (dsDNA), capable of initiating inflammasome assembly upon recognition of foreign or mislocalized DNA in both human and murine systems.^21^^,^^22^

Upon detecting cytosolic dsDNA, AIM2 oligomerizes and recruits’ apoptosis-associated speck-like protein containing a CARD (ASC) and procaspase-1, forming the AIM2 inflammasome complex. This multiprotein assembly activates caspase-1, thereby driving the maturation and secretion of IL-1β and IL-18, and inducing pyroptosis, a lytic form of inflammatory cell death.^23^^,^^24^^,^^25^^,^^26^ Beyond its canonical inflammasome functions, AIM2 has been implicated in the regulation of Tregs stability and function via modulation of cellular metabolic pathways.^27^ This suggests that AIM2 may play a crucial role in shaping Treg development, identity, and immunosuppressive capacity.

Evidence from murine models has shown that AIM2 deficiency increases susceptibility to immune-mediated disorders such as experimental autoimmune encephalomyelitis (EAE) and inflammatory bowel disease, underscoring its essential role in maintaining Treg-mediated immune homeostasis.^28^ However, whether targeting AIM2 within Tregs could disrupt their suppressive functions and thereby enhance antitumor immunity remains unexplored.

In the present study, we developed a Treg-specific AIM2 knockout mouse model to investigate the consequences of AIM2 deficiency on immune equilibrium under steady-state conditions. We further evaluated the therapeutic impact of AIM2-deficient Tregs on the efficacy of ICI in a tumor-bearing setting. Finally, we employed flow cytometry and single-cell RNA sequencing (scRNA-seq) to uncover the cellular and molecular mechanisms underlying these effects.

## Results

### AIM2 deficiency in Tregs does not affect immune homeostasis

To evaluate whether AIM2 deficiency in Tregs disrupted physiological immune equilibrium, we generated a Treg-specific AIM2 knockout mouse line by crossing Foxp3cre mice with Aim2^flox/flox^ mice, producing Aim2^fl/fl^Foxp3^cre^ progeny (Figure 1A). Genomic DNA from tissue samples was analyzed using polymerase chain reaction (PCR) with specific primers to verify the presence of the floxed Aim2 allele and confirm successful Cre-mediated recombination (Figure S1A). In addition, Treg cells were isolated from the spleens and lymph nodes of both groups of mice, and protein extracts were subjected to Western blot analysis (Figure S4G). The results demonstrated a marked reduction of AIM2 protein in Treg cells from the experimental group, confirming the specificity and efficiency of the knockout (Figures S4H–S4I).

**Figure 1:**
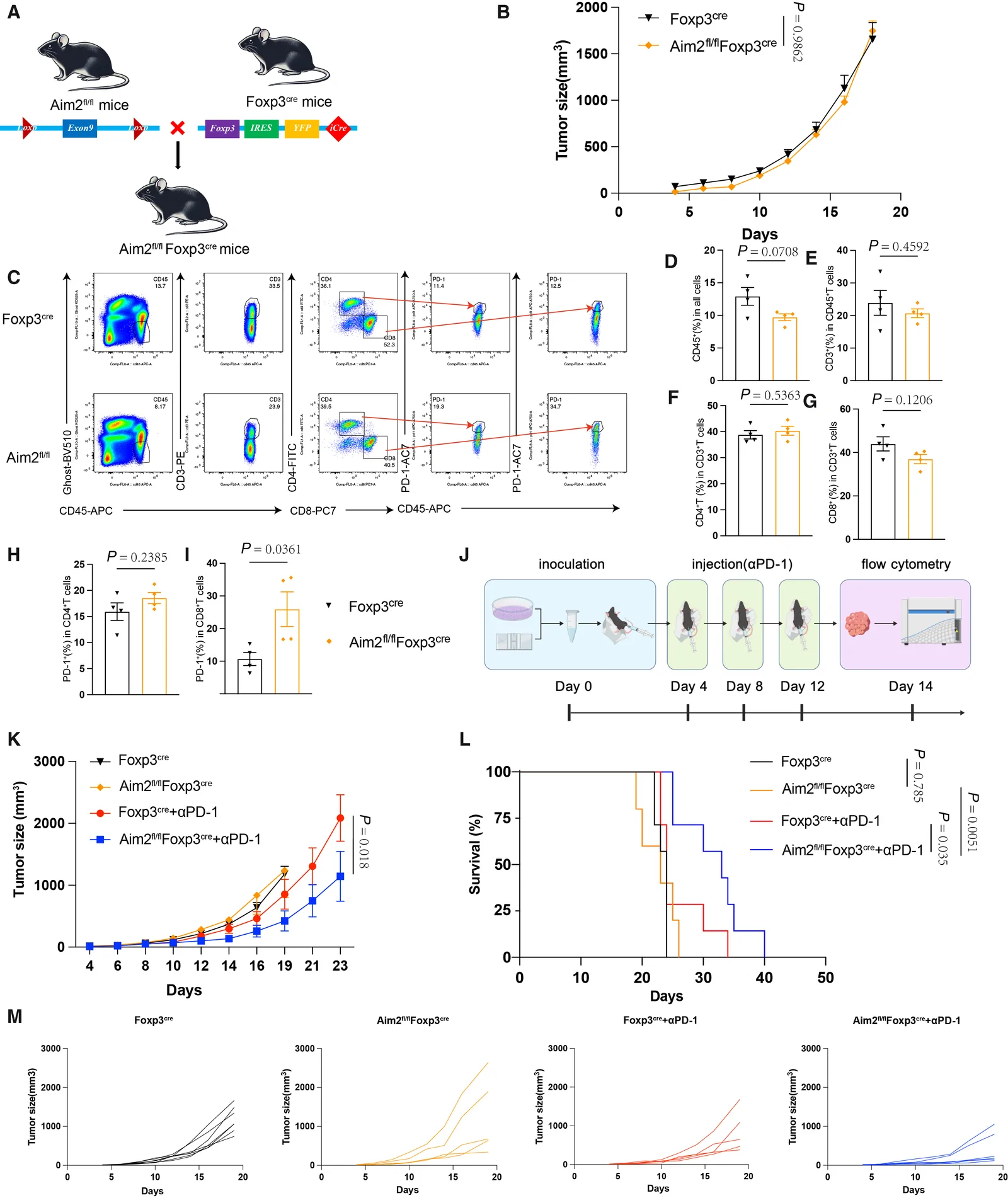
Treg-specific deletion of AIM2 enhances the anti-tumor efficacy of anti-PD-1 therapy 6–8-week-old mice were subcutaneously inoculated with 1 × 10^6^ MC38 colon adenocarcinoma cells per mouse. When the maximum diameter of the tumor reached 5 mm, PD-1 monoclonal antibody treatment (200 μg per mouse) was given every 4 days. Tumors were harvested 48 h after the final treatment (day 14) for flow cytometric analysis (A) Schematic representation of the generation of Aim2^fl/fl^Foxp3^cre^ mice. (B) Tumor growth curves in Foxp3cre and Aim2^fl/fl^Foxp3^cre^ mice without anti-PD-1 treatment (n = 3–4). (C) Representative flow cytometry plots of tumor-infiltrating immune cells from both groups. (D–I) Quantification of the frequency of CD45^+^, CD3^+^, CD4^+^, CD8^+^ T cells, PD-1^+^ CD4^+^ T cells, and PD-1^+^ CD8^+^ T cells (mean ± SEM; *n* = 4; ∗*p* < 0.05). (J) Experimental timeline illustrating tumor model establishment and flow cytometry workflow. (K–L) Tumor growth curves and survival analysis of Foxp3cre, Aim2^fl/fl^Foxp3^cre^, Foxp3^cre^+ αPD-1, and Aim2^fl/fl^Foxp3^cre^ + αPD-1 groups (*n* = 5–7; ∗*p* < 0.05, ∗∗*p* < 0.01). (M) Individual tumor growth curves for each group. Statistical analysis: two-way ANOVA for (B) and (K); Kaplan-Meier survival analysis for (L); two-tailed unpaired *t* test for (E–I).

To assess the impact of AIM2 deficiency in Tregs on organ development and morphology, we examined cohorts of Aim2fl/flFoxp3cre mice at 8 and 20 weeks of age, using age-matched Foxp3cre mice as control. Histological analysis revealed no abnormalities or developmental defects in major organs at either time point (Figures S1B and S1C).

We next explored whether AIM2 deficiency in Tregs influenced the immune architecture of lymphoid organs. Flow cytometry was conducted on spleen, lymph nodes, and thymus from 6-to 8-week-old Aim2fl/flFoxp3cre and Foxp3cre mice, using a standardized gating strategy (Figures S1D and S1E). Quantitative analysis showed no significant differences in the frequencies of CD4^+^ T cells, CD8^+^ T cells, conventional CD4^+^ T cells (CD4^+^Foxp3^-^), or Tregs (CD4^+^Foxp3^+^) between groups (Figures S2A–S2M).

Additionally, we evaluated major myeloid cell populations in the spleen and lymph nodes, including Gr1^+^ myeloid-derived suppressor cells (MDSCs), Gr1^-^ MDSCs, F4/80^+^MHC II^+^ macrophages, myeloid dendritic cells (mDCs), and type I and type II DCs. No appreciable differences were observed between knockout and control mice in any of these subsets (Figures S3A–S3N). Collectively, these findings demonstrated that targeted deletion of AIM2 in Tregs did not perturb immune organ development or alter the composition of key lymphoid and myeloid cell populations under homeostatic conditions.

### Treg-specific ablation of AIM2 enhances the anti-tumor effect of anti-PD-1

To investigate how AIM2 deficiency in Tregs influenced tumor progression, we employed the MC38 murine colon carcinoma model. Tumor growth kinetics showed no significant difference between Aim2^fl/fl^Foxp3^cre^ mice and Foxp3^cre^ controls in the absence of treatment (Figure 1B). Immune profiling of the TME revealed that the overall frequencies of CD45^+^ immune cells, as well as CD3^+^, CD4^+^, and CD8^+^ T cell subsets, remained comparable between the two groups. Notably, however, the proportion of CD8^+^ T cells expressing PD-1 was significantly elevated in tumors from Aim2^fl/fl^Foxp3^cre^ mice relative to controls (Figures 1C–1I).

To assess whether AIM2 deficiency in Tregs modulated the efficacy of ICIs, anti-PD-1 antibody treatment was administered within this tumor model (Figure 1J). Compared to anti-PD-1-treated control mice, Aim2^fl/fl^Foxp3^cre^ mice receiving the same therapy exhibited a slower rate of tumor growth and prolonged survival (Figures 1K–1M). These findings indicated that loss of AIM2 in Tregs potentiated the antitumor activity of PD-1 inhibitor.

### AIM2 deficiency in Tregs contributes to TME remodeling

Our data demonstrated that during the early phase of anti-PD-1 therapy, tumor growth in Aim2^fl/fl^Foxp3^cre^ mice progressively slowed. A statistically significant reduction in tumor size compared to controls was evident 1 week after treatment initiation (Figures 2A–2C).

**Figure 2:**
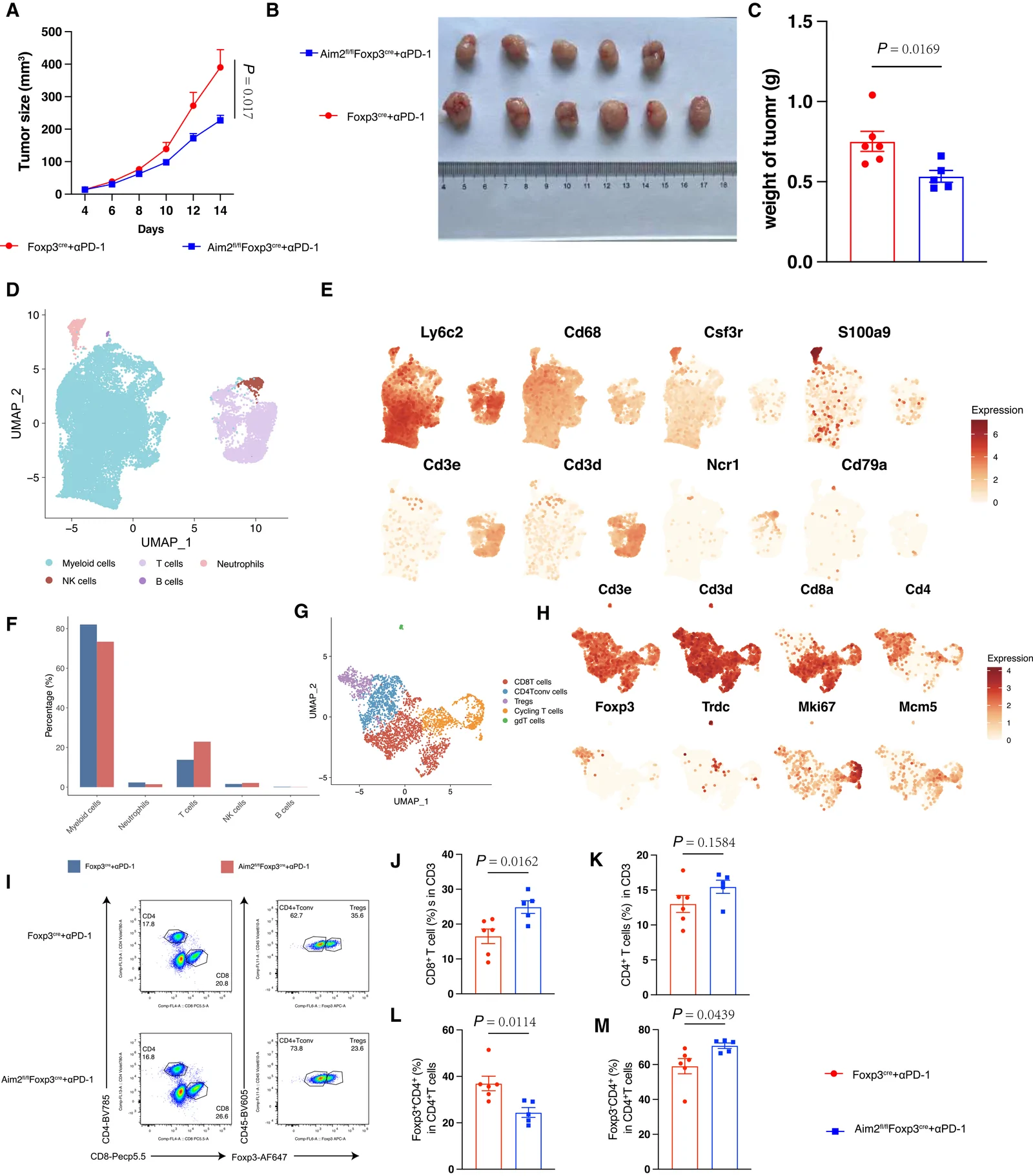
AIM2 deficiency in Tregs remodels the TME (A) Tumor growth curves comparing Foxp3^cre^+ αPD-1 and Aim2^fl/fl^Foxp3^cre^ + αPD-1 mice (*n* = 5–6). (B) Representative images of excised tumors from both groups. (C) Tumor weight quantification (mean ± SEM; *n* = 5–6). (D) UMAP visualization of single-cell transcriptomes from tumor-infiltrating CD45^+^ immune cells, with clusters annotated by immune cell type. (E) UMAP displaying canonical marker gene expression across immune subsets. (F) Bar plot showing proportional changes in immune cell subsets between groups. (G and H) UMAP projections of tumor-infiltrating T cells, showing marker expression patterns defining subpopulations. (I–M) Flow cytometric quantification of CD4^+^ T cells, CD8^+^ T cells, Tregs, and conventional CD4^+^ T cells (mean ± SEM; *n* = 5–6; ∗*p* < 0.05, ∗∗*p* < 0.01, ∗∗∗*p* < 0.001). Statistical analysis: two-way ANOVA for (A); two-tailed unpaired *t* test for (C) and (I–M).

To elucidate the impact of AIM2 deficiency in Tregs on the cellular composition and functional state of the TME, we performed scRNA-seq on CD45^+^ tumor-infiltrating immune cells from both groups. Clustering analysis identified distinct immune populations, which were annotated based on canonical marker expression. These included myeloid subsets (*Ly6c2*, *Cd68*, *Csf3r*, *S100a9*), T lymphocytes (*Cd3e*, *Cd3d*), natural killer (NK) cells (*Ncr1*), and B cells (*Cd79a*) (Figures 2D and 2E), confirming a TME predominantly composed of myeloid and T cells populations.

Under anti-PD-1 treatment, AIM2 deficiency in Tregs led to a marked increase in the relative abundance of T cells, accompanied by a decline in myeloid cell populations (Figure 2F). We further dissected the T cells compartment using established markers, categorizing cells into CD8^+^ T cells, conventional CD4^+^ T cells, Tregs, and proliferative T cells (Figures 2G and 2H). Notably, the proportion of CD8^+^ T cells was significantly elevated in the AIM2-deficient group following anti-PD-1 treatment (Figures 2I and 2J). This finding represents a downstream therapeutic outcome and is conceptually distinct from our earlier observation that, under untreated conditions, CD8^+^ T cells exhibited increased PD-1 expression in the absence of AIM2 in Tregs (Figure 1J). Importantly, the latter observation provided the rationale for anti-PD-1 intervention, linking altered CD8^+^ T cell phenotype to subsequent quantitative improvement upon checkpoint blockade. Although the overall CD4^+^ T cells frequency remained relatively stable, the AIM2-deficient group exhibited a higher percentage of conventional CD4^+^ T cells along with a concomitant reduction in Treg frequency compared to controls (Figures 2K–2M).

### Enhanced CD8 T cell effector function in AIM2 deficient regulatory T cells during checkpoint blockade

To unravel the mechanisms underlying the enhanced antitumor immunity conferred by Treg-specific AIM2 deficiency, we conducted a comprehensive transcriptional profiling of CD8^+^ T cells within the TME, motivated by observed increases in total T cells infiltration and elevated the proportions of CD8^+^ T cells. Utilizing established gene expression signatures, we classified intratumoral CD8^+^ T cells into six distinct transcriptional subsets: naive CD8^+^ T cells, effector CD8^+^ T cells, progenitor exhausted (Tpex) CD8^+^ T cells, terminally exhausted (Tex) CD8^+^ T cells, interferon-γ (IFN-γ)-producing CD8^+^ T cells, and proliferative CD8^+^ T cells (Figures 3A and 3B).

**Figure 3:**
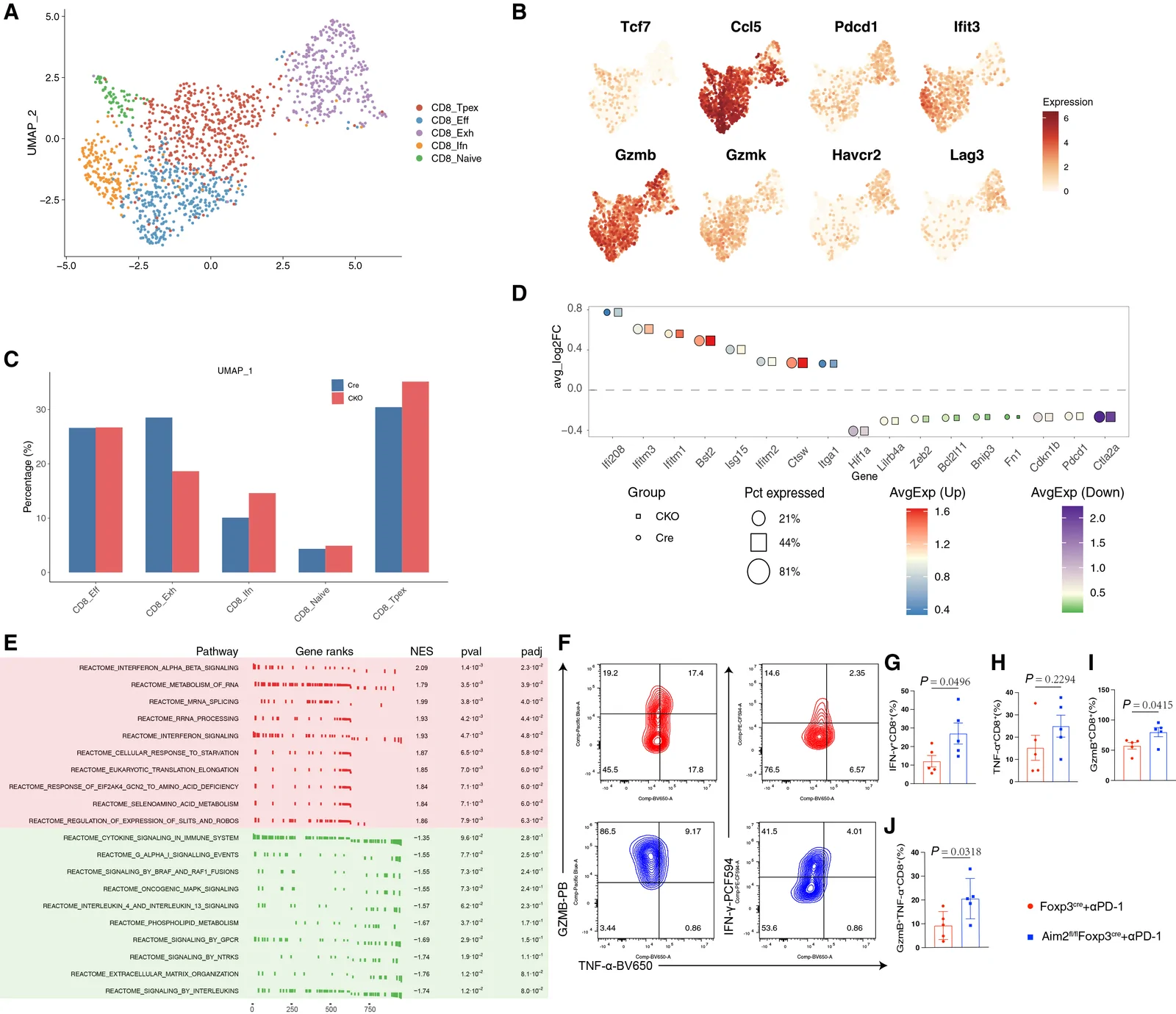
Enhanced CD8^+^ T-cell effector function in AIM2-deficient Tregs during immune checkpoint blockade (A and B) UMAP and dot plot illustrating clustering and canonical marker gene expression of tumor-infiltrating CD8^+^ T cell subsets. (C) Bar plot showing relative proportions of CD8^+^ T cell subsets in Foxp3^cre^ + αPD-1 and Aim2^fl/fl^Foxp3^cre^ + αPD-1 groups, highlighting shifts in immune composition. (D) Volcano plot depicting differentially expressed genes (DEGs) related to tumor immune responses in CD8^+^ T cells within the TME. (E) Reactome pathway enrichment analysis in CD8^+^ T cells; top positively and negatively enriched pathways are ranked by normalized enrichment score (NES). (F–J) Representative flow cytometry plots and corresponding quantifications of IFN-γ, GZMB, and TNF-α expression in tumor-infiltrating CD8^+^ T cells (*n* = 5; mean ± SEM; ∗*p* < 0.05). Statistical analysis: two-tailed unpaired *t* test for (G–J).

Remarkably, Treg-specific AIM2-deficient mice exhibited a relative decrease in effector and Tex subsets, whereas Tpex, IFN-γ-producing, and proliferative CD8^+^ T cells were significantly enriched. This shift reflected a reprogramming of the CD8^+^ T cells compartment toward a less terminally exhausted, more functional, and self-renewing phenotype. Collectively, these alterations indicated that AIM2 loss in Tregs fostered a more immunostimulatory TME capable of sustaining robust CD8^+^ T cells responses, especially under PD-1 inhibitor (Figure 3C).

Differential gene expression analysis of tumor-infiltrating CD8^+^ T cells revealed a transcriptional shift associated with AIM2 deficiency in Tregs (Figure 3D). CD8^+^ T cells exhibited increased expression of interferon-responsive genes, including *Ifitm* family members and Isg15, alongside modest upregulation of effector-associated genes such as *Ctsw* and *Itga1*.

Conversely, inhibitory and stress-related genes, including *Pdcd1*, *Cdkn1b*, and *Hif1a*, were downregulated. Together, these changes indicate transcriptional reprogramming of CD8^+^ T cells toward a pro-inflammatory phenotype, consistent with enhanced antitumor immunity. Gene set enrichment analysis (GSEA) further confirmed robust activation of immune-related pathways within CD8^+^ T cells from Treg-specific AIM2-deficient mice. Key pathways such as interferon α/β signaling, interferon signaling were significantly enriched, underscoring an enhanced antitumor transcriptional program. Simultaneously, pathways involved in RNA processing and protein translation were upregulated, reflecting increased biosynthetic activity characteristic of activated effector cells. In contrast, GSEA revealed a relative downregulation of MAPK-related and other signaling pathways, including BRAF/RAF1 fusion-associated and GPCR-related pathways, in CD8^+^ T cells (Figure 3E). These results point to a shift in the transcriptional landscape of signaling-related genes in CD8^+^ T cells, reflecting altered regulation of intracellular signaling pathways.

To corroborate these transcriptomic findings at the protein level, we performed flow cytometric analysis of tumor-infiltrating CD8^+^ T cells. Consistent with gene expression data, CD8^+^ T cells from Treg-specific AIM2-deficient mice showed significantly higher production of IFN-γ and TNF-α, along with a tendency toward elevated granzyme B (GZMB) expression (Figures 3F–3J). These results validated that AIM2 ablation in Tregs enhanced the functional capacity of CD8^+^ T cells within the TME, thereby improving the efficacy of ICIs therapy.

### Ablation of AIM2 in Tregs undermines their immunosuppressive identity within the TME

Although PD-1 inhibitor potentiated the cytotoxic activity of CD8^+^ T cells, it might paradoxically promote the expansion of immunosuppressive Tregs, which can compromise the overall efficacy of immunotherapy.^29^ Genetic deletion or antibody-mediated inhibition of PD-1 specifically in Tregs has been reported to enhance their proliferation and strengthen their suppressive influence on antitumor immunity.^30^ Guided by these insights, we further interrogated the functional phenotype of AIM2-deficient Tregs within the TME.

Using gene expression profiles, we classified Tregs into three subsets: effector Tregs, IFN-induced Tregs, and proliferative Tregs (Figures 4A and 4B). Notably, AIM2-deficient Tregs exhibited a reduced proportion of effector Tregs, which have been reported to possess potent suppressive capacity, accompanied by an increased fraction of IFN-induced Tregs and a decreased proliferative subset. These compositional changes were associated with a marked reduction in the transcriptional expression of the cytotoxicity-associated marker Gzmb (Figures 4C and 4D), supporting a potential link between AIM2 signaling and Gzmb-associated regulatory programs that may contribute to altered suppressive and proliferative properties of Tregs.

**Figure 4:**
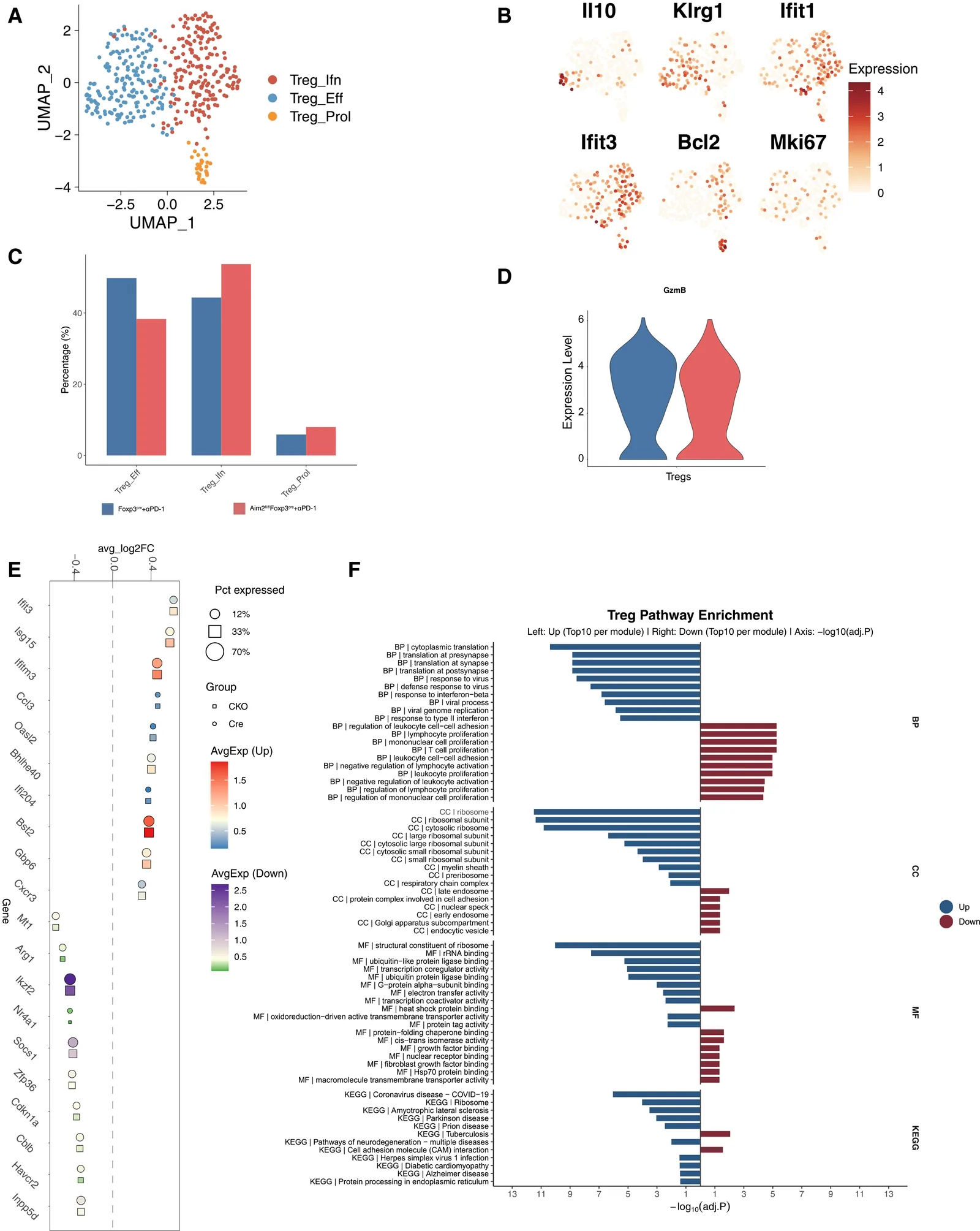
AIM2 deficiency in Tregs disrupts their immunosuppressive phenotype within the TME (A and B) UMAP and dot plot showing clustering and marker gene expression profiles of tumor-infiltrating Treg subsets. (C) Bar plot quantifying relative proportions of Treg subpopulations between experimental groups. (D) Violin plot comparing GZMB expression levels in Tregs across groups (mean ± SEM; ∗∗*p* < 0.01). (E) Dot plot illustrating differentially expressed genes (DEGs) in Tregs. (F) Pathway enrichment analyses (biological gs [mean ± SEM]). Statistical analysis: two-tailed unpaired *t* test for (D).

Differential gene expression analysis revealed that AIM2 deficiency in tumor-infiltrating Tregs was associated with distinct transcriptional changes. A subset of genes related to interferon responsiveness and inflammatory programs, including *Ifit3*, *Isg15*, *Ifitm3*, *Oasl2*, *Bst2*, and *Gbp6*, was upregulated, whereas genes associated with suppressive function and immune regulation, such as *Arg1*, *Ikzf2*, *Nr4a1*, *Socs1*, *Havcr*, and *Inpp5d*, were downregulated (Figure 4E).

Consistent with these gene-level changes, pathway enrichment analysis revealed upregulation of type I and type II interferon responses, viral defense-related pathways, and immune cell proliferation programs, along with pathways involved in ribosomal function and protein processing. These findings suggested that AIM2 deficiency in tumor-infiltrating Tregs not only enhances their inflammatory and antiviral transcriptional programs but may also broadly reprogram cellular metabolism and protein synthesis to support altered immune functions (Figure 4F). However, in a supplementary melanoma B16 model, AIM2 deficiency in Tregs did not affect tumor growth (Figure S4F), suggesting that the efficacy of this intervention may be tumor type dependent.

In line with these transcriptional and pathway-level alterations, we next performed an *in vitro* suppression assay to functionally assess the inhibitory capacity of Tregs in a coculture system with antigen-presenting cells (APCs) and responder T cells (Figure S5A). Tregs, APCs, and responder cells were isolated by flow cytometric sorting and plated using serial dilutions based on a fixed number of Tregs (Figure S5B). The results showed an increase in the number of CD8^+^ T cells in the experimental groups, with the highest CD8^+^ T cell counts observed in wells cultured in the absence of Tregs (Figure S5C). Further analysis revealed that, compared with the control group, coculture with AIM2-deficient Tregs was associated with a reduced proportion of non-proliferating CD8^+^ T cells and a corresponding increase in proliferating CD8^+^ T cells (Figures S5D and S5E), suggesting an attenuation of Treg-mediated suppression of CD8^+^ T cell proliferation.

### AIM2 deficiency in Tregs alters macrophage polarization in the TME

MDSCs contribute significantly to therapeutic resistance, particularly against immunotherapies. Conversely, anti-cancer treatments can modulate MDSCs activity indirectly, either enhancing or suppressing their function.^31^

Using canonical marker genes, myeloid cells were stratified into five subsets: type I tumor-associated macrophages (TAM1), type II tumor-associated macrophages (TAM2), monocytes, DCs, and proliferative macrophages (Figures 5A and 5B). The TME typically skews TAMs toward an M2-like phenotype, which suppresses antitumor immunity.^32^ Remarkably, AIM2 deficiency in Tregs was associated with selective alterations in the myeloid compartment, most prominently reflected by a reduction in the TAM2 population, with a relative increase in TAM1 observed at the single-cell level (Figure 5C). In contrast, changes in monocytes and proliferative macrophages were modest.

**Figure 5:**
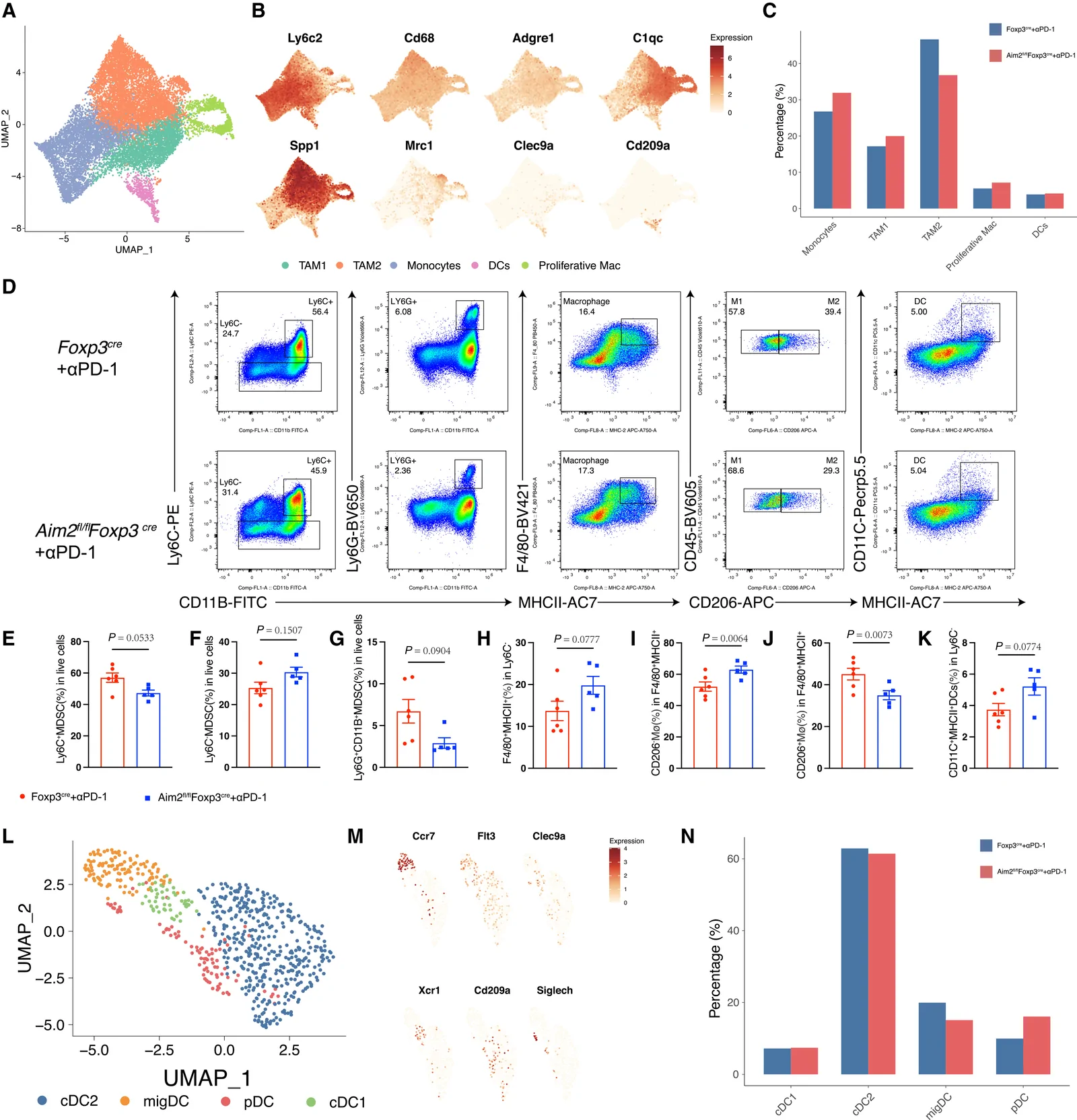
AIM2 deficiency in Tregs modulates macrophage polarization and DC composition within the TME (A and B) UMAP and dot plot depicting clustering of myeloid cell subpopulations and corresponding marker gene expression profiles. (C) Bar graph quantifying the relative composition of myeloid cell subsets in the TME. (D–K) Representative flow cytometry plots and quantification of monocytes, neutrophils, total macrophages, TAM1, TAM2, and DCs in tumor tissues (*n* = 5–6; mean ± SEM; ∗∗*p* < 0.01). (L and M) UMAP and dot plot showing DC subset clustering and marker gene expression. (N) Bar chart illustrating changes in DC subset frequencies between groups. Statistical analysis: two-tailed unpaired *t* test for (E–K).

Consistent with this, flow cytometric analysis revealed comparable overall frequencies of monocytes, neutrophils, and total macrophages between groups, whereas macrophage polarization was selectively affected, with a decrease in TAM2 and a reciprocal increase in TAM1 in Treg-specific AIM2-deficient mice (Figures 5D–5J). Together, these results indicate that AIM2 deficiency in Tregs preferentially influences macrophage polarization—particularly the reduction of immunosuppressive TAM2—rather than inducing broad quantitative remodeling of the myeloid compartment.

Moreover, flow cytometric analysis revealed no substantial changes in the overall abundance of DCs within the TME (Figures 5D and 5K). Consistently, single-cell transcriptomic profiling indicated that the relative frequencies of conventional DC subsets, including cDC1 and cDC2, exhibited only minimal differences between groups, which are unlikely to reflect biologically meaningful alterations in DC composition (Figures 5L–5N).

In contrast, more consistent changes were observed in specific DC subsets. migDCs (migrate DCs), which are involved in antigen transport to draining lymph nodes, showed a reduction, whereas pDCs, a major source of type I interferons, displayed an increase. Together, these findings suggest that AIM2 deficiency in Tregs does not substantially remodel the overall DC landscape but rather is associated with selective shifts in DC subset distribution, particularly affecting migDCs and pDCs. The functional implications of these alterations, including their potential impact on antigen presentation and CD8^+^ T cells responses, warrant further investigation.

### Treg-specific ablation of AIM2 alters intercellular communication among immune subsets following anti-PD-1 therapy

To further characterize intercellular communication within the TME, we performed CellChat analysis based on annotated immune cell populations (Figure 6A). Comparative analysis revealed selective changes in both the number and strength of predicted ligand-receptor interactions following Treg-specific AIM2 deletion (Figure 6B).

**Figure 6:**
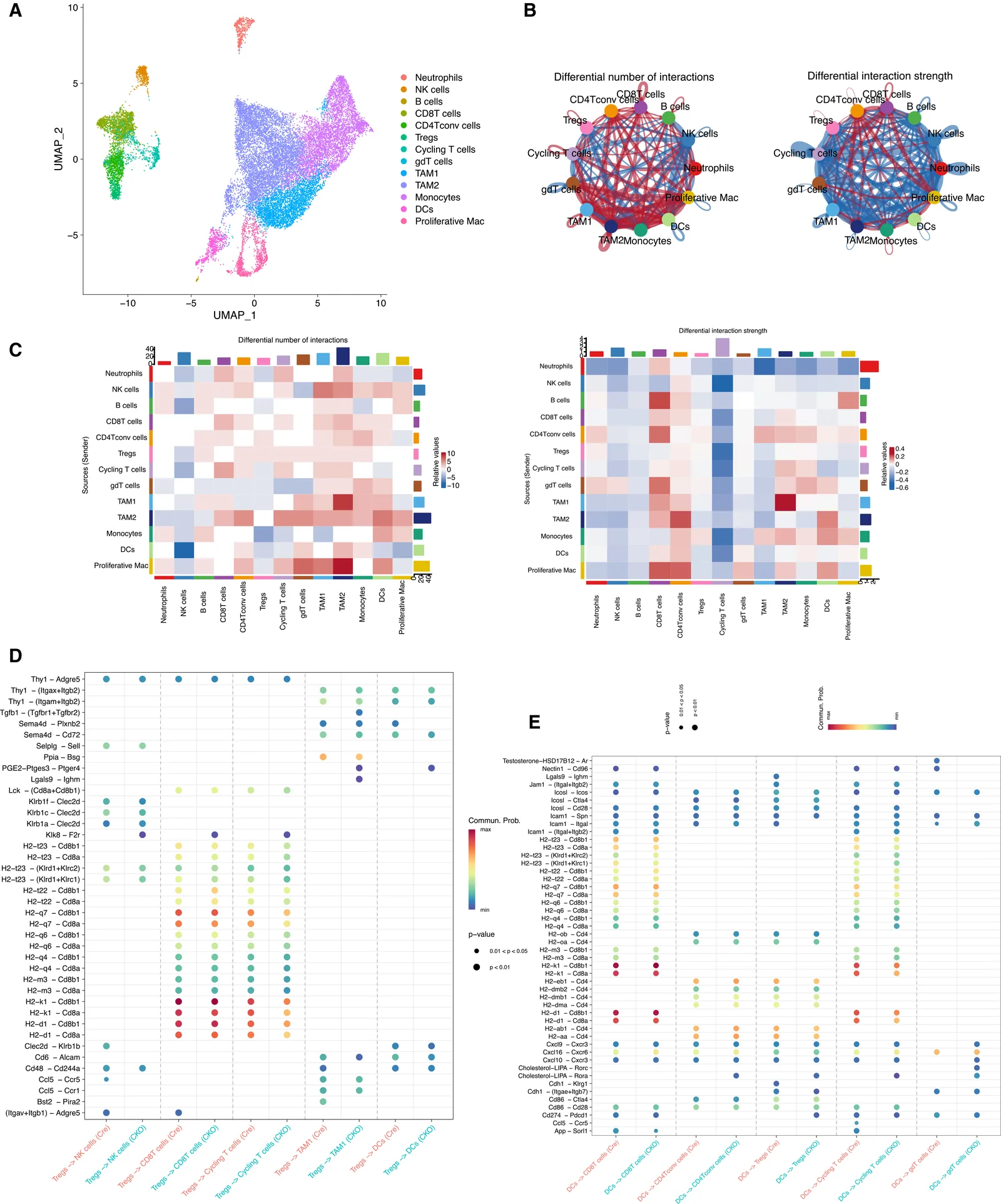
Treg-specific ablation of AIM2 alters intercellular commuication among immune subsets following anti-PD-1 therapy (A) UMAP visualization of tumor-infiltrating immune cells, colored by cell type. (B and C) Interaction network plots (B) and heatmap (C) showing differential interaction strengths among immune cell populations in control and Treg-specific AIM2-deficient mice after PD1 blockade. Blue indicates stronger interactions in control mice, whereas red indicates stronger interactions in AIM2-deficient mice. (D–E) Dot plots showing ligand-receptor interactions between DCs, TAM1 macrophages, and other immune subsets, highlighting altered signaling networks upon AIM2 deficiency in Tregs.Interaction analysis was performed using the CellChat R package.

At the level of individual cell-cell interactions, predicted interactions between Tregs and DCs, as well as between NK cells and DCs, showed a relative reduction, whereas interactions involving conventional CD4^+^ T cells and DCs, together with those between CD8^+^ T cells and TAM1 macrophages, were comparatively increased.

We further examined ligand-receptor interaction patterns involving DCs, TAM1 macrophages, and related immune subsets, and present representative interaction pairs that displayed consistent changes in interaction number and strength (Figures 6C–6E). These included antigen presentation-related interactions (such as H2 family members with CD8a/CD8b1 or CD4), chemokine-associated pairs (such as CXCL9/10-CXCR3 and CXCL16–CXCR6), as well as adhesion- and costimulation-related interactions (including Icam1-and Cd86-associated pairs). In parallel, a subset of regulatory interactions between Tregs and DCs showed reduced interaction levels.

Together, these observations indicate that conditional AIM2 deficiency in Tregs is associated with altered patterns of immune cell communication within the TME.

## Discussion

The current study investigated the pivotal role of AIM2 in modulating antitumor immune responses, focusing on its influence on the efficacy of PD-1 inhibitors through Treg-specific AIM2 deficiency. We demonstrated that ablating AIM2 in Tregs profoundly disrupted their immunosuppressive function, remodeled the TME, and enhanced CD8^+^ T cells effector activity, culminating in marked suppression of tumor growth and extended survival in colorectal cancer models.

Chronic inflammation is a well-recognized driver of tumor progression, with inflammasome receptors playing critical roles in regulating cellular proliferation, gene expression, and oncogenesis. Recent evidence reveals that loss of the AIM2 inflammasome in melanoma modulates tumor growth by interfering with multiple signaling pathways, particularly dampening STING-mediated type I IFN responses.^33^^,^^34^ These findings position AIM2 as a promising molecular target in the context of tumor immunotherapy.

The TME is characterized by intricate cellular interactions, with Tregs orchestrating immunosuppression by inhibiting the activity of effector cells such as CD8^+^ T cells and NK cells, thereby facilitating tumor immune evasion.^35^^,^^36^ Prior research has identified AIM2 as a key regulator of Tregs differentiation and a potential therapeutic target for restoring T cells homeostasis.^27^ This role has been further corroborated in an EAE model, where AIM2 expression is found to be elevated in Tregs. Mechanistically, AIM2 sustains Tregs stability by repressing the Akt-mTOR signaling axis and modulating immune metabolism, thereby mitigating autoimmune disease development.^28^

We engineered a mouse model with selective deletion of AIM2 in Tregs. Although activation of the AIM2 inflammasome is essential for innate immune defense, its dysregulation can lead to excessive inflammation and contribute to autoimmune pathology. AIM2 functions as a crucial regulator of Tregs differentiation and emerges as a potential therapeutic target for restoring T cells homeostasis.^27^^,^^37^ Histopathological examination of multiple organs from Treg-specific AIM2-deficient mice revealed no abnormalities in development or tissue morphology. Moreover, analysis of immune cell subsets within peripheral lymphoid organs, including spleen, lymph nodes, and thymus, showed no significant alterations, indicating that AIM2 loss in Tregs did not perturb overall immune composition or organ integrity.

Considering prior reports highlighting the involvement of AIM2 in CD4^+^ T cells and other immune populations across autoimmune disease models such as experimental autoimmune uveitis (EAU), EAE,^38^^,^^39^ and systemic lupus erythematosus (SLE),^40^ we rigorously assessed the systemic consequences of Treg-specific AIM2 ablation to ensure immunological safety and selectivity. Our data demonstrated that targeted AIM2 deficiency in Tregs effectively dismantled their immunosuppressive phenotype and reprogrammed the TME, thereby potentiating antitumor immune responses.

CD8^+^ T cells are central to mounting effective antitumor immune responses. Nevertheless, during tumor progression, these cells frequently undergo dysfunction or exhaustion, which compromises their capacity to suppress tumor growth.^41^^,^^42^ PD-1 is widely regarded as a hallmark of exhausted CD8^+^ T cells, contributing to the maintenance of their impaired state. However, resistance to PD-1 inhibitor therapies remains a significant barrier limiting their clinical efficacy.^43^^,^^44^ Extensive research efforts have thus focused on strategies to reinvigorate CD8^+^ T cells responses.

In our present study, we observed that AIM2 deficiency in Tregs was associated with increased PD-1 expression on intratumoral CD8^+^ T cells. Based on this observation, we evaluated the combined effect of Treg-specific AIM2 ablation and anti-PD-1 treatment, which was accompanied by enhanced tumor control compared with either condition alone. At the cellular level, loss of AIM2 in Tregs in the context of PD-1 blockade was associated with increased effector-related features of CD8^+^ T cells within the TME. Transcriptional and phenotypic analyses of tumor-infiltrating CD8^+^ T cells revealed changes consistent with a more activated and less exhausted state, including elevated expression of genes related to cytotoxicity, proliferation, and interferon-associated signaling, together with reduced expression of exhaustion-associated markers. Collectively, these observations suggest that AIM2-deficient Tregs exhibit a reduced capacity to restrain CD8^+^ T cell function, which may contribute to improved responses to PD-1 inhibition, although a direct causal relationship cannot be conclusively established.

The TME comprises a complex array of cellular and acellular components that collectively drive tumor progression, invasion, and metastasis and modulate therapeutic responses.^45^ Consistent with expectations, the immunosuppressive capacity of Tregs was markedly diminished in the absence of AIM2 in our model. Within the TME, both effector and proliferative Tregs subsets exhibited a significant reduction. This observation aligned with prior data from the EAE model, where AIM2 deficiency results in decreased Foxp3^+^ Treg populations in the spinal cord and a concomitant increase in IL-17A^+^ and IL-17A^+^IFN-γ^+^ CD4^+^ T cells, indicative of Th17 and Th1/Th17 hybrid phenotypes, underscoring AIM2’s critical role in preserving Treg stability and immune homeostasis.^28^

Furthermore, we observed a pronounced downregulation of GZMB, a pivotal effector molecule expressed by Tregs. Previous studies have implicated GZMB-mediated pathways in Tregs driven tumor immune evasion and metastatic progression.^46^ Thus, diminished GZMB expression in AIM2-deficient Tregs likely alleviated their suppressive impact on antitumor immune effectors, facilitating enhanced tumor cell eradication.

TAMs represent a key cellular component within the TME, profoundly influencing tumorigenesis and resistance to therapy through their immunosuppressive activities.^47^ TAMs are phenotypically heterogeneous, predominantly classified into TAM1 and TAM2 subsets based on their differentiation state and functional properties; TAM1 macrophages exert tumoricidal effects, whereas TAM2 macrophages promote tumor initiation and progression.^48^^,^^49^ IFN-γ secreted by activated CD8^+^ T cells play an instrumental role in recruiting and polarizing macrophages toward the TAM1 phenotype. The reciprocal interaction between cytotoxic CD8^+^ T cells and TAM1 macrophages orchestrates a relay-like cascade of anti-tumor immune responses, a phenomenon amplified during ICIs therapy.^50^^,^^51^

Our study demonstrates that AIM2 restrains antitumor immunity by reinforcing Treg-mediated suppression and shaping the TME. Targeting AIM2 in Tregs offers a potential therapeutic avenue to enhance immune checkpoint blockade efficacy. Beyond mechanistic insight, these findings provide a rationale for developing AIM2-focused interventions as adjuncts to current immunotherapies, with potential to overcome resistance, expand patient responsiveness, and improve clinical outcomes across multiple cancer types.

### Limitations of the study

This study has several limitations that should be acknowledged. Although our findings demonstrate that AIM2 deficiency in Tregs enhances antitumor immunity and improves the efficacy of PD1 blockade, most functional analyses were performed in murine models, and their relevance to human tumors remains to be validated. In addition, the reliance on subcutaneous tumor models does not fully recapitulate the complexity of tissue-specific microenvironments, local invasion, or metastatic progression.

Moreover, while broad remodeling of the tumor immune landscape was observed, the current data do not establish direct causal relationships between Treg-intrinsic AIM2 deficiency and downstream changes in CD8^+^ T cells and myeloid populations. Finally, although AIM2 appears to regulate immunosuppressive programs in Tregs, the precise downstream mechanisms remain incompletely defined, and effective therapeutic strategies targeting AIM2 are currently lacking. Future studies will address these limitations to clarify the mechanistic basis and therapeutic potential of AIM2 in cancer immunotherapy.

## Resource availability

### Lead contact

Requests for further information and resources should be directed to and will be fulfilled by the lead contact, Xiao Zheng (zhengxiao@suda.edu.cn).

### Materials availability

The materials generated in this study are available from the lead contact upon request.

### Data and code availability

• The data of single cell sequencing in this article that support the findings of this study have been deposited into CNGB (https://db.cngb.org/cnsa/) with accession number CNP0009444.
• This article does not report original code.
• Any additional information required to reanalyze the data reported in this article is available from the lead contact upon request.

## Acknowledgments

This work was supported by grants from the 10.13039/501100001809National Natural Science Foundation of China (82303164, 82473269), the Key Project of 10.13039/100017962Jiangsu Provincial Health Commission (K2023069), Jiangsu Provincial Medical Key Discipline (YXZDXK202236), the Science and Technology Support Plan (Social Development) Project of Changzhou (CE20235058, CE20235057).

Technical support in single-cell sequencing was provided by Hangzhou Cosmos Wisdom Biotech Co., Ltd. Particularly grateful are we to YIZEBIOTECH (Changzhou, China) for providing the flow cytometry analysis platform.

## Author contributions

N.W., H.J., Z.F., and J.M. performed the experiments; N.W. and H.J. conducted formal analysis and curated the data; B.X., L.C., S.W., and X.Z. designed the study and contributed to methodology; J.M., B.X., and S.W. provided resources; H.M., B.X., and L.C. performed validation; Z.F., J.M., H.M., and B.X. contributed to project administration; L.C., S.W., and X.Z. acquired funding; N.W. wrote the draft manuscript; H.J., Z.F., J.M., H.M., B.X., L.C., S.W., and X.Z. revised the manuscript. All authors contributed to the article and approved the submitted version.

## Declaration of interests

The authors declare no competing interests.

## STAR★Methods

### Key resources table

**Table:** 

REAGENT or RESOURCE	SOURCE	IDENTIFIER
**Antibodies**		

Anti-mouse CD45 (clone 30-F11)	BioLegend	RRID: AB_2572116
Anti-mouse NK1.1 (clone PK136)	BioLegend	RRID: AB_313394
Anti-mouse CD3 (clone 17A2)	BioLegend	RRID: AB_312677
Anti-mouse CD4 (clone GK1.5) |	eBioscience	RRID: AB_469576
Anti-mouse CD8α (clone 53–6.7)	BioLegend	RRID: AB_312747
Anti-mouse FOXP3 (clone MF-14)	BioLegend	RRID: AB_1089113
Anti-mouse Granzyme B (clone QA16A02)	BioLegend	RRID: AB_2114575
Anti-mouse TNF-α (clone MP6-XT22)	BD Biosciences	RRID: AB_396838
Anti-mouse IFN-γ (clone XMG1.2)	BioLegend	RRID: AB_315402
Anti-mouse F4/80 (clone BM8)	BioLegend	RRID: AB_893478
Anti-mouse Ly6G (clone 1A8)	BioLegend	RRID: AB_2565971
Anti-mouse Ly6C (clone HK1.4)	BioLegend	RRID: AB_2800630
Anti-mouse CD11b (clone M1/70)	BD Biosciences	RRID: AB_2871246
Anti-mouse CD11c (clone N418)	BioLegend	RRID: AB_313777
Anti-mouse CD206 (clone MR6F3)	BioLegend	RRID: AB_10900588
Anti-mouse CD103 (clone M290)	BD Biosciences	RRID: AB_2739520
Anti-mouse PD-1 (J43)	BioXcell	RRID: AB_1107747
Anti-mouse CD3ε/2C11 (clone 17A2)	BioLegend	RRID: AB_2616673
AIM2 Antibody	Cell Signaling	RRID: AB_2732808
Hsp70 Recombinant Rabbit Monoclonal Antibody	HuaBio	RRID: AB_30769593

**Chemicals, peptides, and recombinant proteins**		

DMEM (high glucose)	Gibco (Thermo Fisher Scientific)	Cat# 11965–092
Fetal bovine serum (FBS)	Gibco (Thermo Fisher Scientific)	Cat# 10099–141
Penicillin-Streptomycin (100×)	Gibco (Thermo Fisher Scientific)	Cat# 15140–122
PBS Buffer	Cytiva	Cat# SH30256.01
Trypsin-EDTA (0.25%)	Gibco (Thermo Fisher Scientific)	Cat# 25200–056
DNase I	Sigma-Aldrich (Merck)	Cat# DN25
Liberase TL	Roche	Cat# 05401020001
ACK lysing buffer	Gibco (Thermo Fisher Scientific)	Cat# A10492-01
Intracellular Fixation & Permeabilization Buffer Set	eBioscience	Cat# 88–8824-00
PageRuler™ Plus Prestained Protein Ladder (10–250 kDa)	Thermo Fisher Scientific	Cat# 26619
RIPA lysis buffer	Beyotime Biotechnology	Cat# P0013B
PMSF (Phenylmethylsulfonyl fluoride)	Beyotime Biotechnology	Cat# ST505
Alkaline phosphatase inhibitor cocktail	Beyotime Biotechnology	ST019-10 mM
Bolt™ LDS Sample Buffer (4X)	Thermo Fisher Scientific	Cat# NP0007
NuPAGE™ Transfer Buffer	Thermo Fisher Scientific	Cat# NP0006
Bovine Serum Albumin (BSA)	Solarbio Life Sciences	Cat# SW3015
Tris-buffered saline (TBS), 20×	Solarbio Life Sciences	Cat# T1080
ECL chemiluminescent substrate kit	Solarbio Life Sciences	Cat# SW2040
SafeRed nucleic acid gel stain (10,000×)	Solarbio Life Sciences	Cat# G5590
2× Taq Plus Master Mix II (Dye Plus)	Vazyme	Cat# P213-02
Hematoxylin and Eosin (H&E) staining reagents	Solarbio Life Sciences	Cat# H8070
RPMI1640 (medium)	Gibco (Thermo Fisher Scientific)	Cat# 11875093

**Critical commercial assays**		

FastPure® Tissue DNA Isolation Mini Kit	Vazyme	Cat# DC102-01
CellTrace™ Violet Cell Proliferation Kit	Invitrogen (Thermo Fisher Scientific)	Cat# C34557

**Deposited data**		

RNA-seq	CNGB	CNP0009444

**Experimental models: Cell lines**		

Cell line (Mus musculus): MC38	Cell Bank of the Chinese Academy of Sciences	RRID: CVCL_B288
Cell line (Mus musculus): B16	Cell Bank of the Chinese Academy of Sciences	N/A

**Experimental models: Organisms/strains**		

Mouse: Foxp3^YFP−Cre^	Jackson Laboratory	RRID: IMSR_JAX:016959
Mouse: Aim2^fl/fl^ Foxp3^YFP−Cre^	Jiangsu Jicui Yaokang Biotechnology Co., Ltd.	Strain NO. T009247

**Oligonucleotides**		

JS03123-Aim2-5wt-tF1	CTTGCCAATGTACTCAACAGTTCTC	Primer ID: A2250
JS03123-Aim2-5wt-tR1	AGAAGGTAACAGAGGTGCCATCTAG	Primer ID: A2251
JS03123-Aim2-3wt-tF1	ACAGAGTTCAGGATTTGTAGGATG	Primer ID: A2252
JS03123-Aim2-3wt-tR1	ACCCAACAGTGACTTTGAATATGG	Primer ID: A2253
14125	CCTAGCCCCTAGTTCCAACC	Primer ID: 6206
14126	AAGGTTCCAGTGCTGTTGCT	Primer ID: 6207
16250	AGGATGTGAGGGACTACCTCCTGTA	Primer ID: 5627
16251	TCCTTCACTCTGATTCTGGCAATTT	Primer ID: 5628

**Software and algorithms**		

ImageJ	https://www.imagej.net/	RRID: SCR_003070
Flowjo	https://www.flowjo.com	RRID:SCR_008520
Adobe Illustrator	https://www.adobe.com/products/illustrator.html	RRID:SCR_010279
GraphPad Prism	https://www.graphpad.com/	RRID:SCR_002798
R | The R Project for Statistical Computing	https://www.r-project.org	RRID:SCR_001905
RStudio	https://www.rstudio.com	RRID:SCR_000432

### Experimental model and study participant details

#### Experimental animals and ethical approval

All animal experiments were approved and conducted in strict accordance with protocols approved by the Ethics Committee of Soochow University (Protocol No. (2022) kedi108 at The Third Affiliated Hospital of Soochow University) and in compliance with the National Institute of Environmental Health Sciences/National Institutes of Health guidelines for the humane care and use of laboratory animals. All mouse lines were maintained on a C57BL/6J background under specific-pathogen-free (SPF) conditions at the Cavens Laboratory Animal Center (Changzhou, China). Mice were group-housed with no more than 5 animals per cage under a standard 12-h light/dark cycle and constant temperature, with *ad libitum* access to food and water. Mice between 6 and 8 weeks of age were selected for experimental use.

Treg-specific deletion of AIM2 was achieved by crossing Foxp3Cre mice with Aim2^fl/fl^ mice to generate Treg-specific Aim2 conditional knockout mice (Aim2^fl/fl^Foxp3^c^^re^). Foxp3ˆCre littermates were used as controls. All experimental mice were heterozygous for the transgenes, and sex- and age-matched mice were used. Offspring genotypes were verified via PCR. This breeding strategy allowed for the generation of mice with different genotypes (Aim2^fl/fl^Foxp3^c^^re^ and Aim2^fl/fl^ Cre-negative) for immunological assays, tumor models, and molecular analyses. A schematic overview of the breeding strategy for Aim2^fl/fl^Foxp3^c^^re^ mice is shown in Figure 1.

### Method details

#### Cell culture and tumor models

MC38 murine colorectal adenocarcinoma cells were obtained from the Shanghai Institute of Life Sciences, Chinese Academy of Sciences, and were routinely tested to confirm the absence of mycoplasma contamination. Cells were cultured in Dulbecco’s Modified Eagle Medium (DMEM), supplemented with 10% fetal bovine serum (FBS) and 1% penicillin-streptomycin (P/S), all purchased from Gibco (Thermo Fisher Scientific), and maintained at 37°C in a humidified incubator containing 5% CO_2_.

Foxp3^Cre^ and Aim2^fl/fl^ mice on a C57BL/6 background were maintained under specific pathogen-free (SPF) conditions with *ad libitum* access to food and water under a 12 h light/dark cycle. Male mice between 6–8 weeks of age were used in all experiments (Figure S1C shows H&E sections of 20-week-old male mice). All animal procedures were approved by the Institutional Animal Care and Use Committee of The Third Affiliated Hospital of Soochow University (Protocol No. [2022] kedi108) and conducted in accordance with institutional guidelines and NIH recommendations.

For tumor implantation, 1 × 10^6^ MC38 cells suspended in 50 μL sterile PBS were subcutaneously injected into the abdominal flanks of mice. Tumor growth was monitored periodically using calipers, and tumor volume was calculated using the formula: volume = (length × width^2^)/2.

For anti–PD-1 treatment, mice received antibody therapy beginning 4–5 days after tumor inoculation. Anti–PD-1 monoclonal antibody (clone J43; Bio X Cell) was diluted in sterile phosphate-buffered saline (PBS) and administered intraperitoneally at a dose of 200 μg per mouse in a total volume of 100 μL. Subsequent treatments were performed every 4 days using the same dose and route until experimental endpoints were reached.

#### PCR (polymerase chain reaction)

Ear and tail tissue samples from mice were used for genomic DNA extraction, which was performed using the FastPure Blood/Cell/Tissue/Bacteria DNA Isolation Mini Kit (Vazyme Biotech Co., Ltd., Nanjing, China), in accordance with the supplier’s protocol. PCR was conducted on a T100 Thermal Cycler (Bio-Rad Laboratories) using the 2× Taq Plus Master Mix II (Dye Plus) from Vazyme. The PCR mixture had a total volume of 25 μL and consisted of the following components: 1 μL of extracted genomic DNA, 1 μL each of the forward and reverse primers, 12.5 μL of 2× Taq Plus Master Mix, and 9.5 μL of nuclease-free water. After an initial denaturation at 95°C for 5 min, the amplifications were carried out with 36 cycles at a melting temperature of 95°C for 30 s, an annealing temperature of 58°C for 30 s, and an extension temperature of 72°C for 30 s, followed by a final extension at 72°C for 3 min. The reaction was then cooled to room temperature (25°C) for temporary storage.

PCR products were resolved on a 1.5% agarose gel at 120 V for 30 min, stained with SolarRed Nucleic Acid Dye (10,000×) (Beijing Solarbio Science & Technology Co., Ltd, Beijing, China), and visualized under UV illumination. Primers were designed and synthesized by Sangon Biotech (Shanghai) Co., Ltd (Shanghai, China). Detailed primer sequences are provided in key resources table-Oligonucleotides.

#### Hematoxylin and eosin (H&E) staining

Organs were collected from Foxp3^cre^ and Aim2^fl/fl^ mice at 8 and 20 weeks of age and immediately fixed in 4% paraformaldehyde (PFA) at 4 °C overnight. Following fixation, tissues were dehydrated through a graded ethanol series, cleared with xylene, and embedded in paraffin blocks. Serial 5-μm sections were prepared using a rotary microtome and mounted on glass microscope slides.

For H&E staining, paraffin-embedded sections were first deparaffinized in xylene and rehydrated through a descending ethanol gradient into distilled water. Sections were stained with hematoxylin for 5 min, rinsed under running tap water, and differentiated in 1% acid alcohol. After bluing in 0.1% ammonia water, the sections were counterstained with eosin for 2 min. Slides were then dehydrated through ascending ethanol concentrations, cleared again in xylene, and sealed with coverslips using a synthetic resin mounting medium.

Bright field scanning of stained slides was performed by Wuhan Shunbai Biotechnology Co., Ltd. (Wuhan, China).

#### Flow cytometry

Tumor dissociation and flow cytometric staining were performed with detailed optimization as described below. Tumor specimens were collected 24–48 h after anti–PD-1 treatment and immediately transferred into 6-well culture plates containing serum-free RPMI-1640 medium. Tissues were mechanically minced into small fragments and enzymatically digested at 37 °C for 30 min in digestion buffer consisting of 0.25 mg/mL Liberase TL (Roche) and 0.33 mg/mL DNase I (Sigma-Aldrich). Digested samples were subsequently filtered through a 70-μm cell strainer to obtain single-cell suspensions.

Cells were washed and resuspended in RPMI-1640 medium supplemented with 1% fetal bovine serum (FBS) for all antibody dilutions and staining procedures. Cell viability was assessed using Ghost Dye™ Violet 510 (Cell Signaling Technology) to discriminate live from dead cells.

For surface staining, single-cell suspensions derived from tumor tissues were incubated with fluorochrome-conjugated antibodies diluted at 1:100, whereas intracellular antibodies (including Foxp3 and CD206) were diluted at 1:60. For immune cells isolated from peripheral lymphoid organs, surface antibodies were used at a dilution of 1:300, and intracellular antibodies (including Foxp3 and CD206) were diluted at 1:100. All antibodies used are listed in the key resources table.

For intracellular cytokine detection, cells were stimulated with a leukocyte activation cocktail (BioLegend) at 37 °C for 6 h prior to fixation and permeabilization using the FOXP3/Transcription Factor Staining Buffer Set (eBioscience). Cells were then stained for intracellular IFN-γ and TNF-α according to the manufacturer’s instructions.

Flow cytometric acquisition was performed on a CytoFLEX flow cytometer (Beckman Coulter), and data were analyzed using FlowJo software (version 10.8.1).

#### scRNA-seq data processing

Raw sequencing data generated by the 10× Genomics platform were initially processed using the Cell Ranger pipeline, with reads aligned to the mm10 reference genome for quantification of unique molecular identifiers (UMIs). The resulting gene expression matrices were then imported into the Seurat R package (version 4.3.0.1) to construct Seurat objects for downstream single-cell analysis. Quality control was conducted using the DoubletFinder package, evaluating key parameters such as the total number of UMIs, the percentage of mitochondrial gene expression per cell, and the number of detected genes (nFeature_RNA). Cells were filtered based on stringent criteria: ≥ 6,000 detected genes, ≥ 30,000 total UMIs (nCount_RNA), and ≤ 5% mitochondrial gene content.

After quality filtering, dimensionality reduction was carried out using principal component analysis (PCA), followed by uniform manifold approximation and projection (UMAP) to visualize cellular heterogeneity. Clustering was performed according to the standard Seurat workflow, and distinct immune cell populations were identified based on canonical marker genes established in our previous studies.

#### Western blot analysis

Protein samples were extracted using RIPA lysis buffer supplemented with protease and phosphatase inhibitors. After centrifugation at 12,000 rpm for 20 min at 4 °C, the supernatants were collected and denatured with loading buffer by boiling at 95°C–100°C for 5–10 min. Equal amounts of protein were separated by SDS–PAGE and subsequently transferred onto polyvinylidene difluoride (PVDF) membranes using a wet transfer system.

Membranes were blocked with 5% bovine serum albumin (BSA) or non-fat dry milk in TBST for at least 1 h at room temperature and then incubated overnight at 4 °C with primary antibodies diluted according to the manufacturers’ instructions. After washing with TBST, membranes were incubated with appropriate horseradish peroxidase (HRP)-conjugated secondary antibodies for 1 h at room temperature. Immunoreactive bands were visualized using enhanced chemiluminescence (ECL) reagents and imaged with a chemiluminescence detection system. Band intensities were quantified using ImageJ software, and target protein expression was normalized to α-tubulin as a loading control.

#### *In vitro* treg suppression assay

Treg cells were isolated from tumors and Responder cells, and antigen-presenting cells (APCs) were isolated from spleens of control and experimental mice, all prepared as single-cell suspensions. CD45^+^ immune cells were first enriched using anti-mouse CD45 magnetic beads and columns, followed by flow cytometric sorting to obtain Treg cells from tumors and APCs and Responder cells from spleens. Sorted cells were maintained in complete RPMI 1640 medium supplemented with 10% FBS and 1% penicillin–streptomycin to preserve viability.

Responder cells were washed with PBS, resuspended in 2 mL PBS, and labeled with CellTrace Violet at a 1:1000 dilution. Cells were incubated in a 37 °C water bath for 10 min, and staining was terminated by adding culture medium. Cell numbers were determined under a microscope and adjusted to defined concentrations: Treg, 4000 events/100 μL (first column); Responder cells, 4000 events/50 μL; APCs, 16,000 events/50 μL.

In 96-well U-bottom plates, 50 μL RPMI 1640 was added to each well except for the first column. Treg cells (100 μL) were added to the first column, then 50 μL from each well of the first column was transferred sequentially to the second and third columns to achieve a gradient dilution of Treg cells. Subsequently, 50 μL of CellTrace-labeled Responder cells and 50 μL APCs were added to each well, followed by 50 μL anti-CD3 monoclonal antibody (2C11, 4 μg/mL) to activate T cells. Plates were gently mixed, covered, and incubated at 37 °C with 5% CO_2_ for 72 h, with PBS added to empty wells to maintain humidity.

After co-culture, cells were transferred to V-bottom plates and centrifuged at 1500 rpm for 5 min. Cells were then stained with appropriate antibodies, prepared as samples, and analyzed by flow cytometry.

### Quantification and statistical analysis

All statistical analyses and data visualizations were performed using GraphPad Prism software (version 10.3.1, build 464, RRID:SCR_002798). Experimental variables are presented as mean ± standard error of the mean (SEM). Two-group comparisons were assessed using two-tailed unpaired Student’s t-tests. For tumor growth kinetics, statistical differences were evaluated using two-way analysis of variance (ANOVA). Exact *p* values are reported in the figure legends.
