# Supplementary material for: AIM2 in tregs restrains anti-PD-1 efficacy by reinforcing immune suppression in MC38 colorectal cancer
Source: iScience. 2026 Sep 24;29(10):116082. doi: 10.1016/j.isci.2026.116082 (PMC13634901; doi:10.1016/j.isci.2026.116082)
Supplement: Supplementary file 1 [file mmc1.pdf]

iScience, Volume *29*

Supplemental information

AIM2 in tregs restrains anti-PD-1 efficacy by reinforcing immune suppression in MC38 colorectal cancer

Nuo Wang, Hongwei Jiang, Zhang Fang, Jiajin Ma, Hui Ma, Bin Xu, Lujun Chen, Shaoxian Wu, and Xiao Zheng

Figure S1

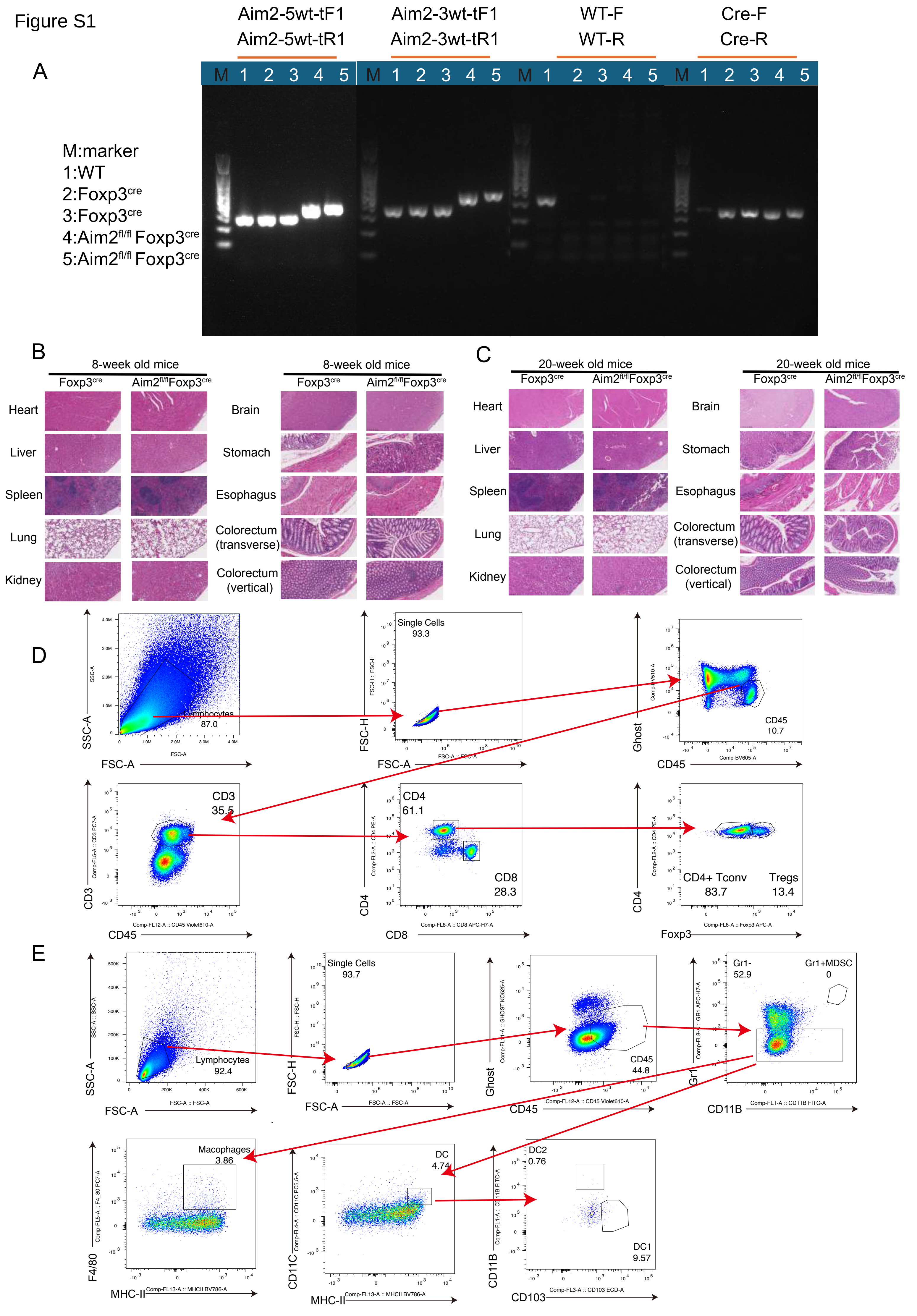

A
Aim2-5wt-tF1
Aim2-5wt-tR1
Aim2-3wt-tF1
Aim2-3wt-tR1
WT-F
WT-R
Cre-F
Cre-R
M 1 2 3 4 5
M:marker
1:WT
2:Foxp3cre
3:Foxp3cre
4:Aim2fl/fl Foxp3cre
5:Aim2fl/fl Foxp3cre
B
8-week old mice
Foxp3cre
Aim2fl/flFoxp3cre
Heart
Liver
Spleen
Lung
Kidney
Brain
Stomach
Esophagus
Colorectum (transverse)
Colorectum (vertical)
C
20-week old mice
D
Lymphocytes 87.0
Single Cells 93.3
CD45 10.7
CD3 35.5
CD4 61.1
CD8 28.3
CD4+ Tconv 83.7
Tregs 13.4
SSC-A
FSC-A
FSC-H
Ghost
CD45
CD3
CD4
CD8
Foxp3
E
Lymphocytes 92.4
Single Cells 93.7
CD45 44.8
Gr1- 52.9
Gr1+MDSC 0
Macophages 3.86
DC 4.74
DC2 0.76
DC1 9.57
Gr1
CD11B
F4/80
MHC-II
CD11C
CD103

Figure S1 AIM2 deficiency in Tregs does not induce pathological alterations.

(A) PCR genotyping of wild-type, *Foxp3*^{cre}, and *Aim2*$^{fl/fl}$*Foxp3*^{cre} mice.

(B–C) Representative H&E-stained sections of heart, liver, spleen, lung, and kidney from 8-week-old (B) and 20-week-old (C) *Foxp3*^{cre} and *Aim2*$^{fl/fl}$*Foxp3*^{cre} mice, showing no histopathological abnormalities.

(D) Flow cytometry gating strategy for identification of T cells populations.

(E) Flow cytometry gating strategy for myeloid cell population identification.

Figure S2

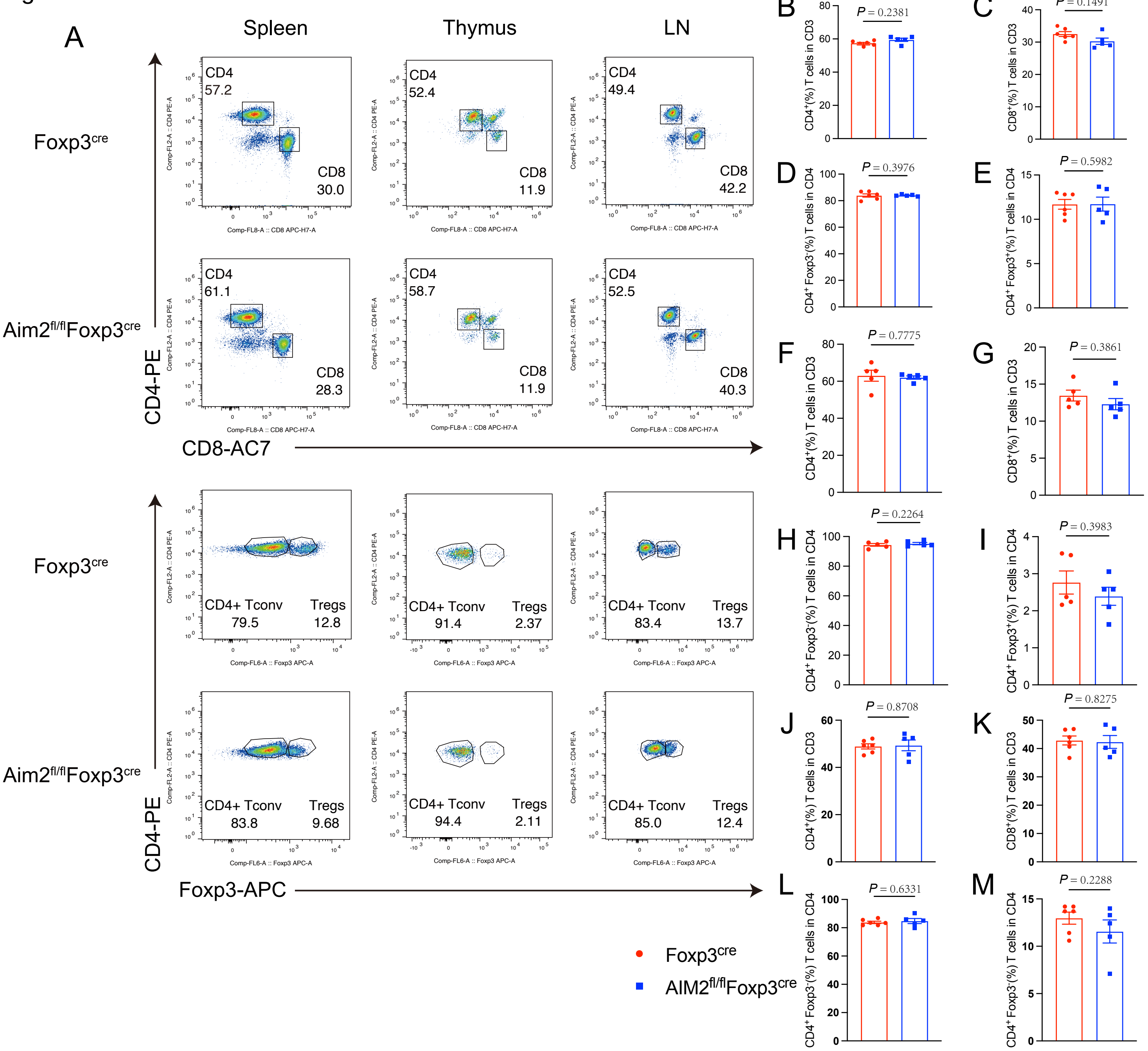

Figure S2 AIM2 deficiency in Tregs does not alter T cells compartment composition.

(A) Representative flow cytometry plots of T cells populations in spleen, thymus, and lymph nodes from *Foxp3*^{cre} and *Aim2*$^{fl/fl}$*Foxp3*^{cre} mice.

(B–E) Quantification of CD4^{+} T cells, CD8^{+} T cells, Tregs, and conventional CD4^{+} T cells in the spleen.

(F–I) Quantification of the same T cells subsets in the thymus.

(J–M) Quantification of the same T cells subsets in lymph nodes. Data are shown as mean ± SEM (n = 5–6). No significant differences were observed (ns, $P > 0.05$; two-tailed t-test).

Figure S3

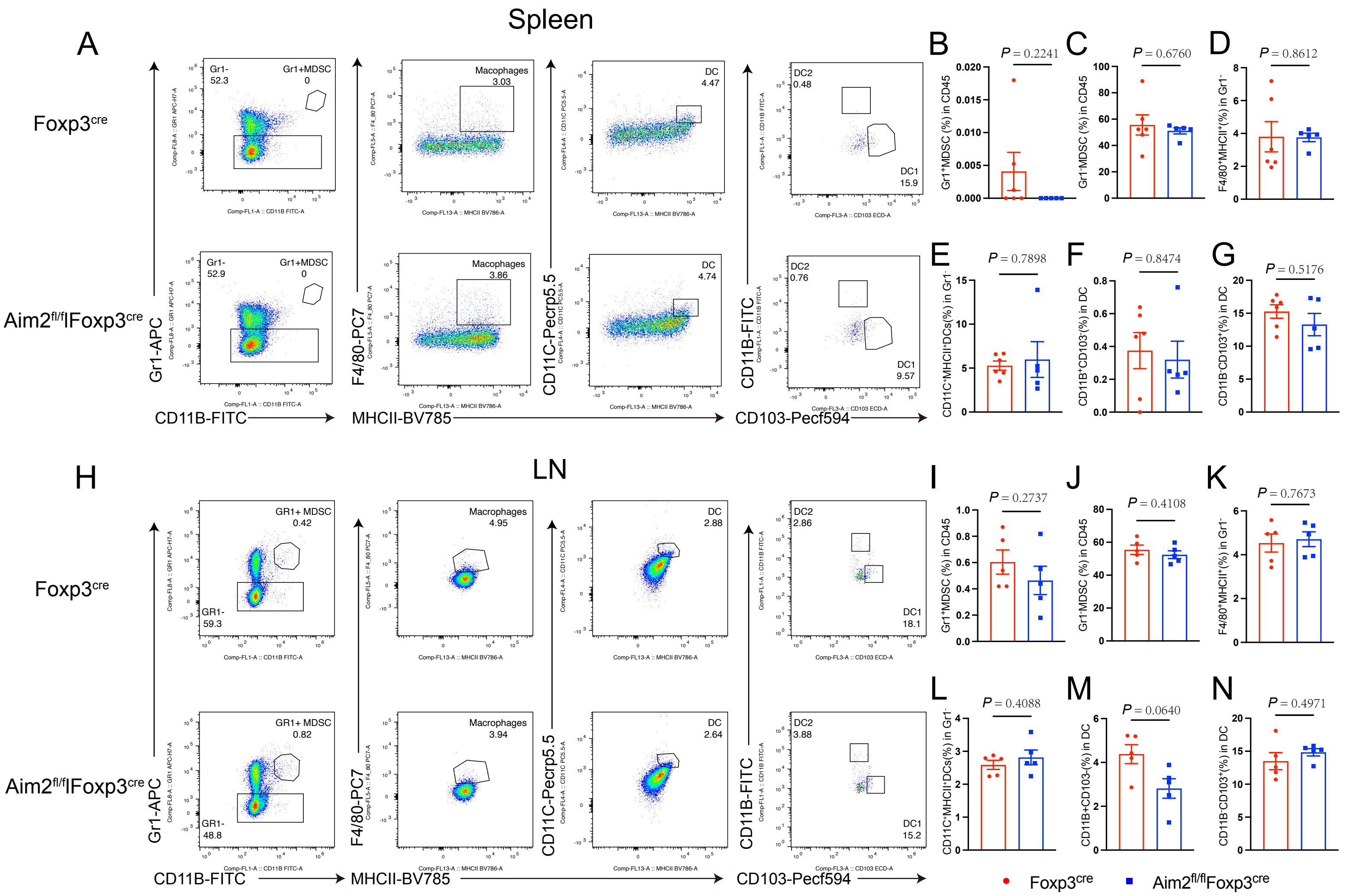

Figure S3 AIM2 deficiency in Tregs minimally impacts myeloid cell populations.

(A) Representative flow cytometry plots of myeloid populations in spleen from *Foxp3^cre^* and *Aim2^fl/fl^Foxp3^cre^* mice.

(B–G) Quantification of Gr1^{+} MDSCs, Gr1^{-} MDSCs, macrophages, DCs, cDC1, and cDC2 in the spleen.

(H) Representative flow cytometry plots of myeloid populations in lymph nodes.

(I–N) Quantification of the same myeloid subsets in lymph nodes. Data are shown as mean ± SEM (n = 5–6). No significant differences were observed (ns, *P* > 0.05; two-tailed t-test).

Figure S4

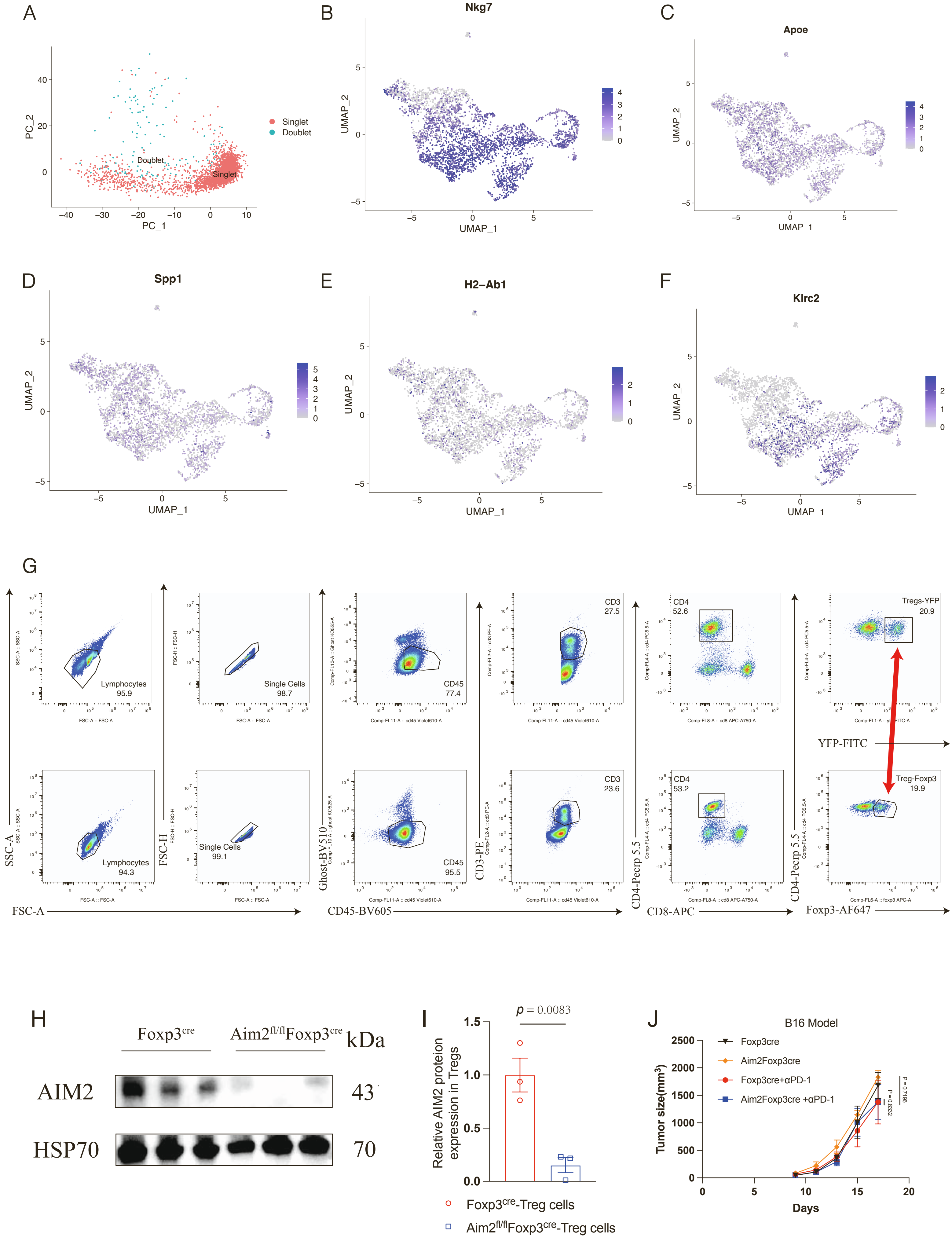
A
PC_2
PC_1
Singlet
Doublet
B
Nkg7
UMAP_2
UMAP_1
C
Apoe
D
Spp1
E
H2–Ab1
F
Klrc2
G
Lymphocytes 95.9
Single Cells 98.7
CD45 77.4
CD3 27.5
CD4 52.6
Tregs-YFP 20.9
YFP-FITC
Lymphocytes 94.3
Single Cells 99.1
CD45 95.5
CD3 23.6
CD4 53.2
Treg-Foxp3 19.9
SSC-A
FSC-H
Ghost-BV510
CD3-PE
CD4-Pecrp 5.5
FSC-A
CD45-BV605
CD8-APC
Foxp3-AF647
H
Foxp3cre
Aim2fl/flFoxp3cre
kDa
AIM2
43
HSP70
70
I
p = 0.0083
Relative AIM2 proteion expression in Tregs
Foxp3cre-Treg cells
Aim2fl/flFoxp3cre-Treg cells
J
B16 Model
Foxp3cre
Aim2Foxp3cre
Foxp3cre+αPD-1
Aim2Foxp3cre +αPD-1
Tumor size(mm3)
Days
P = 0.7196
P = 0.8332

Figure S4 Sorting of Treg cells and expression of AIM2.

(A–F) Representative workflow for the sequential depletion of double-positive cells and re-selection of the $CD8^+$ cell population.

(G) YFP fluorescence versus intracellular staining for Treg sorting.

(H) Western blot analysis of protein extracts from Treg cells sorted from mouse spleen and lymph nodes.

(I) Quantification of Western blot band intensities shown as a bar graph. (n = 3; $^{**}P < 0.01$; two-tailed t-test).

(J) Tumor growth curves in the subcutaneous B16 melanoma model

Figure S5

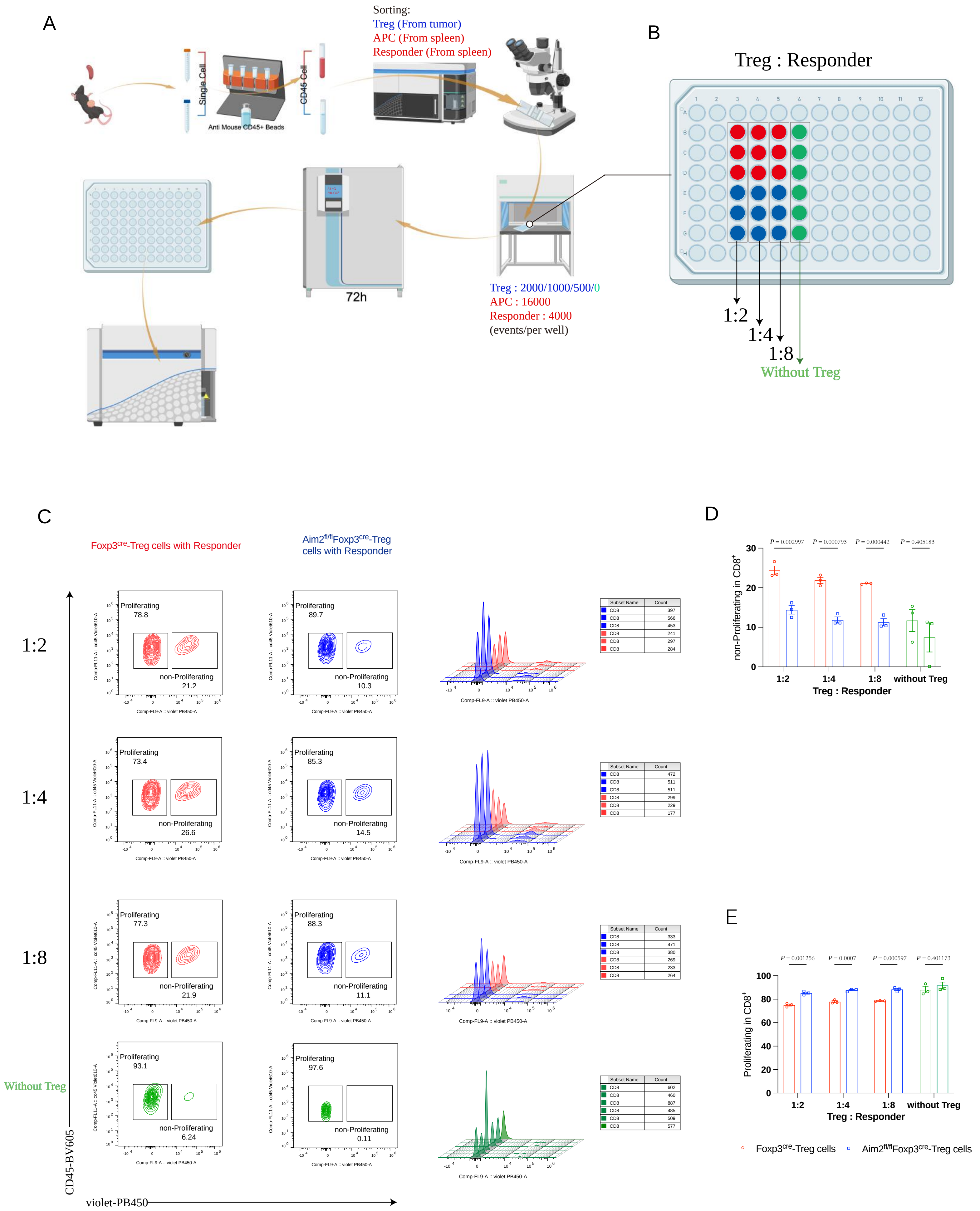
A
Sorting:
Treg (From tumor)
APC (From spleen)
Responder (From spleen)
Single Cell
CD45 Cell
Anti Mouse CD45+ Beads
72h
Treg : 2000/1000/500/0
APC : 16000
Responder : 4000
(events/per well)
B
Treg : Responder
1:2
1:4
1:8
Without Treg
C
Foxp3cre-Treg cells with Responder
Aim2fl/flFoxp3cre-Treg cells with Responder
1:2
1:4
1:8
Without Treg
Proliferating
78.8
non-Proliferating
21.2
Proliferating
89.7
non-Proliferating
10.3
Proliferating
73.4
non-Proliferating
26.6
Proliferating
85.3
non-Proliferating
14.5
Proliferating
77.3
non-Proliferating
21.9
Proliferating
88.3
non-Proliferating
11.1
Proliferating
93.1
non-Proliferating
6.24
Proliferating
97.6
non-Proliferating
0.11
Comp-FL11-A :: cd45 Violet610-A
Comp-FL9-A :: violet PB450-A
Subset Name
Count
CD8
397
566
453
241
297
284
472
511
511
299
229
177
333
471
380
269
233
264
602
460
887
485
509
577
CD45-BV605
violet-PB450
D
P = 0.002997
P = 0.000793
P = 0.000442
P = 0.405183
non-Proliferating in CD8+
Treg : Responder
1:2
1:4
1:8
without Treg
E
P = 0.001256
P = 0.0007
P = 0.000597
P = 0.401173
Proliferating in CD8+
Treg : Responder
1:2
1:4
1:8
without Treg
Foxp3cre-Treg cells
Aim2fl/flFoxp3cre-Treg cells

Figure S5 Treg in vitro inhibitory function test.

(A) Schematic representation of the complete workflow for the in vitro Treg suppression assay.

(B) Layout of 96-well plates used for in vitro co-culture. The A-B images were drawn using BioGDP (*https://biogdp.com*)

(C) Flow cytometric analysis of CellTrace-labeled responder cells after 72 h of co-culture, showing cell proliferation.

(D–E) Quantification of flow cytometry results presented as bar graphs. (n = 3; ns, $P > 0.05$, $^{*}P < 0.05$, $^{**}P < 0.01$, $^{***}P < 0.001$; two-tailed t-test)

Table S1

AIM2	Absent in Melanoma 2
ANOVA	Analysis of Variance
AOPI	Acridine Orange/Propidium Iodide
ASC	Apoptosis-associated speck-like protein containing a CARD
BD	Becton Dickinson (BD Biosciences)
BM8	Anti-F4/80 monoclonal antibody clone BM8
BRAF	B-Raf proto-oncogene, serine/threonine kinase
CARD	Caspase Activation and Recruitment Domain
CCL8	C-C Motif Chemokine Ligand 8
cDC1	Conventional Dendritic Cell Type 1
cDC2	Conventional Dendritic Cell Type 2
CXCL10	C-X-C Motif Chemokine Ligand 10
CXCR3	C-X-C Chemokine Receptor 3
CXCR6	C-X-C Chemokine Receptor 6
DC	Dendritic Cell
DMEM	Dulbecco’s Modified Eagle Medium
DNA	Deoxyribonucleic Acid
EAU	Experimental Autoimmune Uveitis
EAE	Experimental Autoimmune Encephalomyelitis
FBS	Fetal Bovine Serum
Foxp3	Forkhead box P3
GSEA	Gene Set Enrichment Analysis
GZMB	Granzyme B
H&E	Hematoxylin and Eosin
ICIs	Immune Checkpoint Inhibitors
IFN	Interferon
IFN-γ	Interferon gamma
IL	Interleukin
Lag3	Lymphocyte Activation Gene 3
MDSC	Myeloid-Derived Suppressor Cell
MC38	Murine Colon Adenocarcinoma cell line 38
mDC	Myeloid Dendritic Cell
NK	Natural Killer cell
Ncr1	Natural Cytotoxicity Triggering Receptor 1
PCR	Polymerase Chain Reaction
PD-1	Programmed Death-1
PD-L1	Programmed Death-Ligand 1
pDC	Plasmacytoid Dendritic Cell
RNA	Ribonucleic Acid
scRNA-seq	Single-cell RNA sequencing
SEM	Standard Error of the Mean

SLE	Systemic Lupus Erythematosus
SPF	Specific Pathogen-Free
TAM1	Tumor-Associated Macrophage Type 1
TAM2	Tumor-Associated Macrophage Type 2
Tconv	Conventional T cell
Tex	Terminally Exhausted T cell
TIL	Tumor-Infiltrating Lymphocyte
TME	Tumor Microenvironment
TNF-α	Tumor Necrosis Factor-alpha
Tpex	Progenitor Exhausted T cell
Treg	Regulatory T cell
UMAP	Uniform Manifold Approximation and Projection
